# Expanding cross-presenting dendritic cells enhances oncolytic virotherapy and is critical for long-term anti-tumor immunity

**DOI:** 10.1038/s41467-022-34791-8

**Published:** 2022-11-22

**Authors:** Judit Svensson-Arvelund, Sara Cuadrado-Castano, Gvantsa Pantsulaia, Kristy Kim, Mark Aleynick, Linda Hammerich, Ranjan Upadhyay, Michael Yellin, Henry Marsh, Daniel Oreper, Suchit Jhunjhunwala, Christine Moussion, Miriam Merad, Brian D. Brown, Adolfo García-Sastre, Joshua D. Brody

**Affiliations:** 1 Hematology and Medical Oncology, Icahn School of Medicine at Mount Sinai, New York, NY 10029 USA; 2 Division of Molecular Medicine and Virology, Department of Clinical and Experimental Medicine, Linköping University, Linköping, 582 25 Sweden; 3 Tisch Cancer Institute, Icahn School of Medicine at Mount Sinai, New York, NY 10029 USA; 4 Department of Microbiology, Icahn School of Medicine at Mount Sinai, New York, NY 10029 USA; 5 Celldex Therapeutics, Inc, Needham, MA 02494 USA; 6 Genentech, South San Francisco, CA 94080 USA; 7 Department of Oncological Sciences, Icahn School of Medicine at Mount Sinai, New York, NY 10029 USA; 8 The Precision Immunology Institute, Icahn School of Medicine at Mount Sinai, New York, NY, 10029 USA; 9 Department of Genetics and Genomic Sciences, Icahn School of Medicine at Mount Sinai, New York, NY 10029 USA; 10 Global Health Emerging Pathogens Institute, Icahn School of Medicine at Mount Sinai, New York, NY 10029 USA; 11 Department of Medicine, Division of Infectious Diseases, Icahn School of Medicine at Mount Sinai, New York, NY 10029 USA; 12 Present Address: Department of Hepatology and Gastroenterology, Campus Virchow- Klinikum, Charité Universitätsmedizin Berlin, Berlin, 13353 Germany

**Keywords:** Cancer immunotherapy, Tumour vaccines, Antigen-presenting cells

## Abstract

Immunotherapies directly enhancing anti-tumor CD8^+^ T cell responses have yielded measurable but limited success, highlighting the need for alternatives. Anti-tumor T cell responses critically depend on antigen presenting dendritic cells (DC), and enhancing mobilization, antigen loading and activation of these cells represent an attractive possibility to potentiate T cell based therapies. Here we show that expansion of DCs by Flt3L administration impacts in situ vaccination with oncolytic Newcastle Disease Virus (NDV). Mechanistically, NDV activates DCs and sensitizes them to dying tumor cells through upregulation of dead-cell receptors and synergizes with Flt3L to promote anti-tumor CD8^+^ T cell cross-priming. In vivo, Flt3L-NDV in situ vaccination induces parallel amplification of virus- and tumor-specific T cells, including CD8^+^ T cells reactive to newly-described neoepitopes, promoting long-term tumor control. Cross-presenting conventional Type 1 DCs are indispensable for the anti-tumor, but not anti-viral, T cell response, and type I IFN-dependent CD4^+^ Th1 effector cells contribute to optimal anti-tumor immunity. These data demonstrate that mobilizing DCs to increase tumor antigen cross-presentation improves oncolytic virotherapy and that neoepitope-specific T cells can be induced without individualized, ex vivo manufactured vaccines.

## Abstract (web summary)

Strategies to advance T cell based immune therapies are mostly focusing on the improvement of CD8 T cell effector functions, such as cytotoxicity or recruitment to the tumor. Here authors show that by combining in situ vaccination with oncolytic Newcastle Disease Virus and Flt3L-driven dendritic cell expansion, the anti-tumor T cell response is amplified via increased antigen cross-presentation.

## Introduction

Most cancer immunotherapies depend on the induction of tumor-specific CD8^+^ T cells recognizing tumor antigens (Ag) presented on class I MHC. Therefore, much effort has been made to enhance these T cell responses at the effector phase, e.g. increasing activating signals such as IL-2/7/15 or by blocking inhibitory signals with checkpoint blockade^1^. While these strategies have been successful for a subset of patients, they are limited by the requirement of pre-existing tumor-specific T cells. An alternative to enhancing anti-tumor T cells is to harness the potential of dendritic cells (DC) to induce anti-tumor T cells de novo by cross-presenting tumor Ag and providing T cell co-stimulatory signals. Cross-presenting DCs are not only important for the initiation of T cell responses but also for the development of long-lasting T cell memory, and most immunotherapies (e.g. checkpoint blockade, adoptive T cell transfer, vaccines) fail completely in the absence of cross-presenting DCs^2–6^. In patients with advanced-stage cancer, expanding intratumoral (i.t.) DCs can promote systemic tumoral CD8^+^ T cell infiltration and long-lasting tumor regressions^7^. Efficient anti-tumor T cell responses require that DCs are mobilized, loaded with tumor Ag, and activated, but how this is best achieved remains an open question.

A critical step is loading DCs with Ag to achieve optimal tumor Ag-specific CD8^+^ T cell responses. Recent advances have enabled definition of potential tumor Ag with individualized tumor exome and RNA sequencing, neoepitope prediction, peptide synthesis and subcutaneous administration^8^. Such ex vivo produced vaccines have induced neoepitope-reactive T cells in early trials but are both time and resource intense. Long manufacturing time is a limitation that may preclude this approach for e.g. rapidly growing tumors. Likewise, resource intensity is a challenge as studies demonstrate that socioeconomic status and health-care inequalities affect the accessibility to immunotherapies^9,10^, highlighting the need of treatment strategies that are not only efficient but also accessible to a large number of patients. An alternative, less time and resource intense, approach is to release tumor Ag and activate DCs at the site of a patient’s tumor, i.e. in situ vaccination (ISV), which eliminates the need to pre-define tumor Ag. We have previously shown that effective ISV can be achieved using Flt3-ligand (Flt3L) to mobilize DCs, radiotherapy to release tumor Ag and load DCs, and TLR agonists (TLRa) to activate Ag-loaded DCs^7^. Although a wide array of TLRa have been investigated clinically^11^, some yielding tumor regressions^7,12–14^, the optimal strategy to activate DCs that induce efficient anti-tumor T cell immunity is unknown. One promising ISV strategy is the use of oncolytic viruses that can both induce immunogenic cell death to load DCs with tumor Ag and stimulate pro-inflammatory signals to activate DCs^15^. While the loss of DCs abrogates the efficacy of oncolytic virotherapy^16,17^, it is unknown whether increasing i.t. DCs will potentiate oncolytic virus-based ISV. Here, we used Newcastle disease virus (NDV), a non-human pathogen with well-documented oncolytic activity, safety profile and demonstrable, but moderate, clinical efficacy in a wide variety of cancers^18,19^. NDV, being a negative sense single-stranded RNA virus, has the advantage of inducing a robust type I interferon (IFN) response in mammals^20,21^.

In this study, we present a detailed characterization of the immune response to NDV and the impact of increasing i.t. DCs by administration of Flt3L. Further, to enable rigorous assessment of oncolytic virus-induced anti-tumor T cell immunity, we used lymphoma as cancer model because of its unique sensitivity to various T cell-mediated immunotherapies. Using murine and patient-derived DCs, we demonstrate that DC activation and tumor Ag uptake induced by NDV is substantially increased by Flt3L. Flt3L-NDV ISV results in improved T cell cross-priming, durable tumor regressions and the induction of tumor-specific T cells that persist long-term. Using exome and RNA sequencing we identify neoepitope candidates and show that Flt3L-NDV ISV induces neoepitope-reactive CD8^+^ T cells, ultimately accomplishing a similar result to neoepitope vaccines, but with an off-the-shelf approach.

## Results

### NDV enhances immunogenicity and susceptibility of tumor cells to T cell-mediated killing

**Fig. 1:**
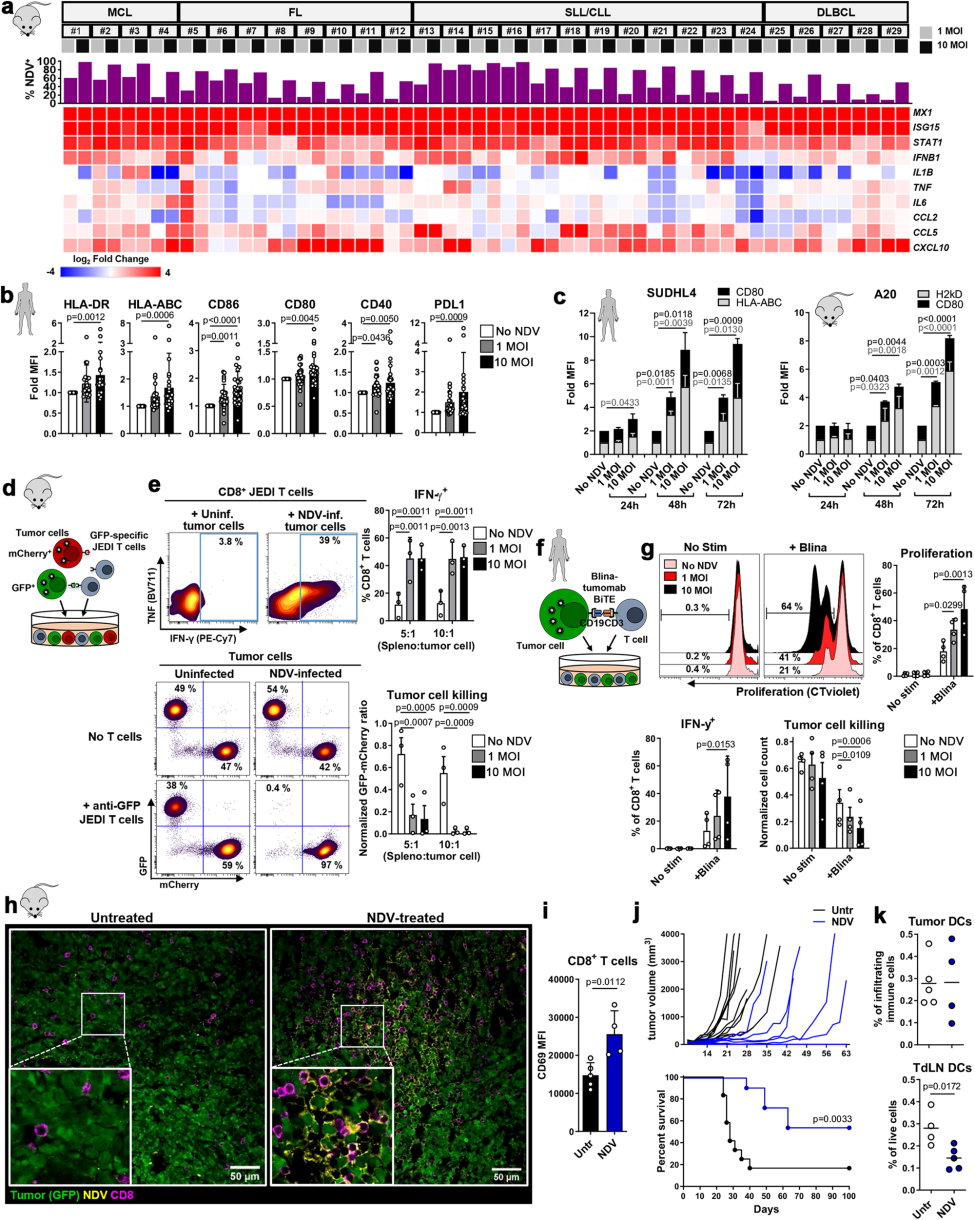
NDV enhances immunogenicity and susceptibility of tumor cells to T cell-mediated killing. **a** Expression of IFN-stimulated and pro-inflammatory genes in patient lymphoma samples (MCL and SLL/CLL from blood, FL and DLBCL from lymph nodes) 24 h post infection (p.i.) with NDV; top graph shows percent infected CD19^+^ tumor (analyzed by flow cytometry); the heat map shows the Log_2_ fold expression vs ‘No NDV’ (quantitative RT-PCR) (*n* = 29). **b**, **c** Fold expression (vs ‘No NDV’) of MHC and co-stimulatory molecules on (**b**) patient lymphoma cells (*n* = 29) 24 h p.i. and (**c**) SUDHL4 (HLA-ABC, *n* = 4, CD80, *n* = 6) and A20 (H2kD, *n* = 2, CD80, *n* = 3) cells 24, 48 and 72 h p.i. Repeated measures One-way ANOVA (**b**) or Two-way ANOVA (**c**) with Dunnett’s multiple comparisons test. **d**, **e** Uninfected/NDV-preinfected GFP^+^ and mCherry^+^ A20 cells (ratio 1:1) were co-cultured with JEDI splenocytes at the indicated ratios. JEDI CD8^+^ T cell activation and tumor cell killing (**e**) were analyzed after 5 days (*n* = 3). Repeated measures Two-way ANOVA with Dunnett’s multiple comparisons test. **f** Uninfected/NDV-preinfected SUDHL4 cells were co-cultured with CD8^+^ T cells in the presence of Blinatumomab (Blina). T cell activation and tumor cell killing (**g**) were analyzed after 3 days (*n* = 4). Repeated measures One-way ANOVA with Dunnett’s multiple comparisons test. **h** GFP^+^ A20 tumor-bearing Balb/c mice were treated with intratumoral NDV and tumors were harvested after 24 h. Representative confocal images are shown (Untr, *n* = 2; NDV, *n* = 3). **i** Intratumoral CD8^+^ T cells from mice treated as in (**h**) were analyzed by flow cytometry (Untr, *n* = 5; NDV, *n* = 4; unpaired, two-tailed t-test). **j** GFP^+^ A20 tumor-bearing mice were treated with NDV (days 8, 10, 12, 14) and monitored for tumor growth and survival (untreated, *n* = 12; NDV, *n* = 11). Log-rank (Mantel-Cox) test. **k** Intratumoral and TdLN DCs from mice treated as in (**h**) were analyzed by flow cytometry (Untr, *n* = 5; NDV, *n* = 4;, unpaired, two-tailed t-test). Data show mean ± SD. MCL mantle cell lymphoma, SLL small lymphocytic lymphoma, CLL chronic lymphocytic leukemia, FL follicular lymphoma, DLBCL diffuse large B cell lymphoma.

B-cell lymphomas, like other tumors, downregulate MHC and co-stimulatory molecules to evade immune recognition^22^, and are generally resistant to checkpoint blockade therapy. To assess the ability of NDV to enhance antigen presentation and immune activation, we measured the infectivity of NDV in multiple lymphoma subtype patient samples and its effect on expression of pro-inflammatory genes and MHC and co-stimulatory molecules. Whereas NDV infected different lymphoma subtypes with variable efficiency (Fig. 1a), it induced robust expression of IFN-stimulated genes (*STAT1, MX1*, *ISG15*) across all patient samples (Fig. 1a). Further, NDV induced expression of *CCL5* and *CXCL10*, implicated in T cell recruitment and associated with responses to immunotherapy^23^. Because the source of the induced genes is unclear, as patient samples contain both tumor and immune cells, we also analyzed the direct effects of NDV in human (SUDHL4) and mouse (A20) B cell lymphoma cell lines. NDV infection resulted in dose-dependent cell death and reduced tumor cell numbers over time (Supplementary Fig. 1a) and the induction of IFN-stimulated genes, similar to lymphoma patient samples (Supplementary Fig. 1b) and consistent with previous studies on bladder and melanoma lines^24^. Furthermore, NDV induced the upregulation of MHC I/II, co-stimulatory molecules (CD40, CD80, CD86) and PDL1 in patient samples, with increased expression over time (Fig. 1b and Supplementary Fig. 1c), with similar effects on SUDHL4 and A20 cell lines (Fig. 1c). These data suggest that in addition to direct tumor killing, NDV may improve tumor Ag recognition and enhance T cell-mediated cytotoxicity.

To test this, we used GFP as a surrogate tumor Ag and co-cultured naïve anti-GFP JEDI CD8^+^ T cells^25^ with uninfected or NDV-preinfected mixtures of GFP^+^ and mCherry^+^ A20 cells (Fig. 1d). Co-culture with NDV-preinfected tumor cells resulted in increased proliferation, activation and IFN-γ production by JEDI CD8^+^ T cells (Fig. 1e and Supplementary Fig. 1d) and greatly enhanced tumor Ag-specific T cell killing, as seen by marked decrease in the GFP:mCherry cell ratio (Fig. 1e). Similar effects were observed when free NDV was removed post tumor infection, prior to splenocyte co-culture, indicating that direct infection of immune cells is dispensable for the observed enhanced T cell tumor-killing (Supplementary Fig. 1e). Notably, even low-dose NDV which infects only a minority of tumor cells (~20%, Supplementary Fig. 1a) enables anti-GFP T-cell killing of >99% of GFP^+^ A20 cells, indicating potentiation of Ag-specific killing even in non-infected cells. Further, co-culture with NDV-infected tumor cells resulted in the production of TNF and IFN-γ by (GFP-non-specific) CD4^+^ T cells (Supplementary Fig. 1f), possibly induced in an Ag-independent or virus Ag-specific manner and mediated by cytokines such as IFN I^26,27^, which may further amplify the activation of tumor Ag-specific CD8^+^ T cells. To assess whether virus:tumor:T cell interactions are also observed in human cells, we co-cultured NDV-preinfected SUDHL4 cells with human T cells with or without the bispecific T cell engager (BiTE) Blinatumomab, inducing contact-mediated killing through binding of CD19 on the tumor cells and CD3 on T cells (Fig. 1f). Similar to the murine system, NDV enhanced CD8^+^ T cell activation, and that of CD4^+^ T cells, per their increased proliferation and IFN-γ production, and increased sensitivity of tumor cells to contact-mediated T cell killing (Fig. 1g and Supplementary Fig. 1g).

The in vitro NDV-enhanced T cell activation and tumor killing prompted us to assess NDV anti-tumor effects in vivo. We treated established, subcutaneous GFP^+^ A20 lymphoma tumors with i.t. NDV injection and harvested these 24 h later, revealing NDV viral proteins within GFP^+^ A20 cells, indicating substantial infection (Fig. 1h). There was also accumulation of CD8^+^ T cells in NDV-rich areas (Fig. 1h), paralleled by widespread i.t. caspase 3 activation (Supplementary Fig. 1h) and significant increase in the early activation marker CD69 on i.t. CD8^+^ T cells (Fig. 1i). Continued treatment resulted in initial tumor growth delay or regression, however, a substantial proportion of mice had late tumor relapses (Fig. 1j), indicating that oncolytic therapy alone may not efficiently promote long-term tumor control.

### Flt3L enhances cross-priming of anti-tumor CD8^+^ T cells upon NDV-cytolysis

**Fig. 2:**
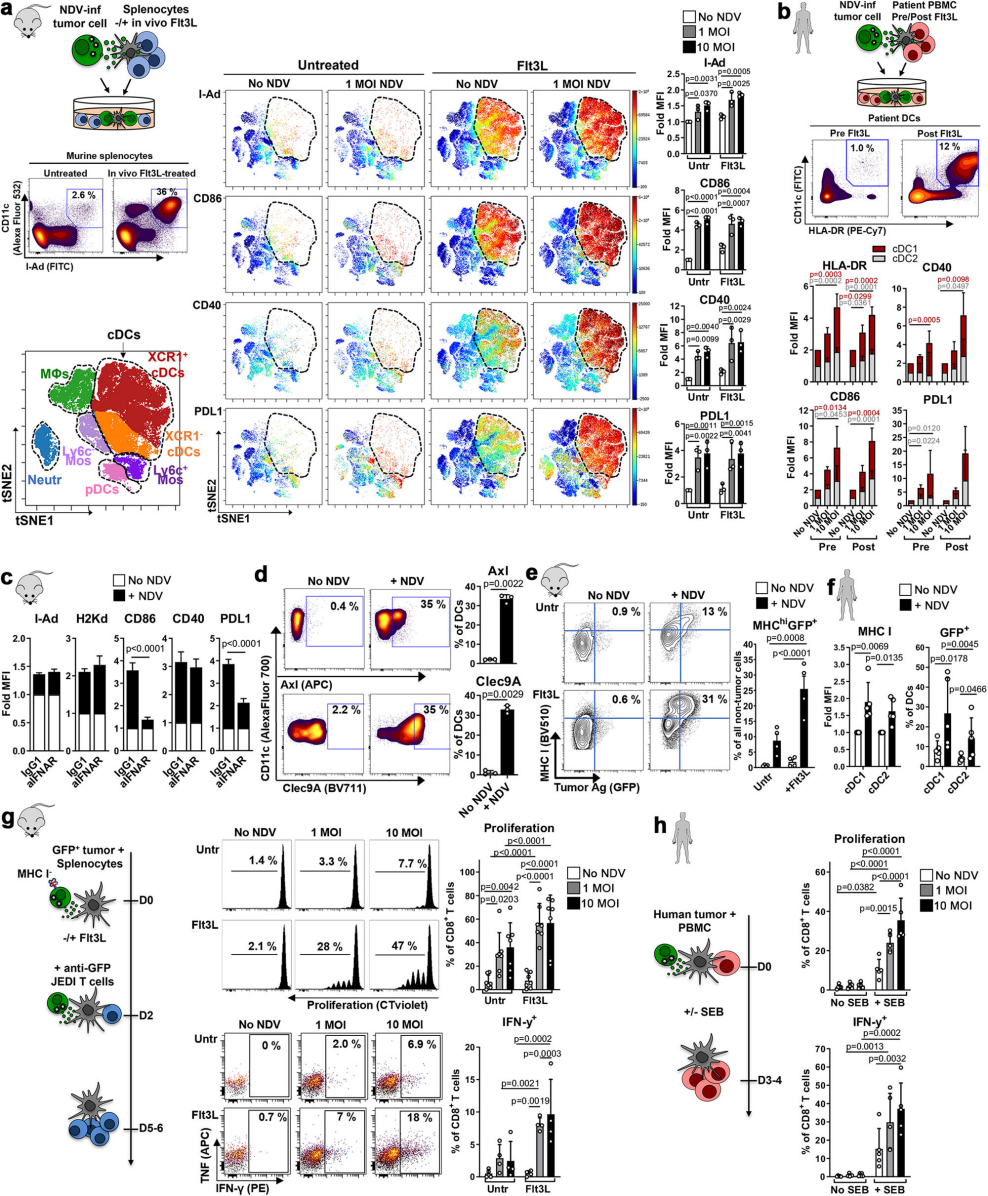
Flt3L enhances cross-priming of anti-tumor CD8^+^ T cells upon NDV-cytolysis. **a** Uninfected/NDV-preinfected GFP^+^ A20 cells were co-cultured with splenocytes from untreated/Flt3L-treated mice (contour plots show % cDCs of all splenocytes) and analyzed after 24 h. Representative viSNE plots showing myeloid cell populations (left) and relative expression (color code = mean MFI) of different markers (right). Bar graphs show mean MFI of cell-surface markers on cDCs (CD11c^+^I-Ad^+^). Repeated measures two-way ANOVA with Dunnett’s multiple comparisons test. *n* = 3 **b** Uninfected/NDV-preinfected GFP^+^ SUDHL4 cells were co-cultured with patient-derived PBMCs, pre- and post-Flt3L treatment, and analyzed after 24 h. Representative (*n* = 5) contour plots showing percent HLA-DR^+^CD11c^+^ cDCs (of all non-tumor cells) pre- and post-Flt3L treatment. Stacked bar graphs showing fold expression (vs ‘No NDV’) of activation markers in cDC1s (CD141^+^) and cDC2s (CD1c^+^). One-way ANOVA with Dunnett’s multiple comparisons test (*n* = 5). **c** Cells were cultured as in (**a**) with IFNAR-blocking or isotype-control antibodies and cDCs were analyzed. **d** Expression of Axl and Clec9A in cDCs cultured as in (**a**). **c**, **d** Graphs show No NDV vs 10 MOI data analyzed in triplicates, representative from at least 2 independent experiments. One-way ANOVA with Dunnett’s multiple comparisons test. **e** Tumor Ag (GFP)-uptake and MHC I expression in splenocytes from untreated/Flt3L-treated mice, after co-culture with uninfected/NDV-preinfected GFP^+^ A20 cells (*n* = 4); repeated measures One-way ANOVA with Sidak’s multiple comparisons test. **f** Tumor Ag (GFP)-uptake and MHC I expression in human cDC1 (CD141^+^) and cDC2 (CD1c^+^) DCs in PBMCs derived from Flt3L-treated patients, after co-culture with uninfected/NDV-preinfected GFP^+^ SUDHL4 cells. Repeated measures One-way ANOVA with Dunnett’s multiple comparisons test (*n* = 5). **g** NDV-preinfected MHC I-deficient GFP^+^ A20 cells were co-cultured with splenocytes (from untreated or Flt3L-treated mice) for 48 h and CellTrace violet-stained JEDI T cells were added to co-cultures and analyzed after 3–4 days. Representative flow cytometry data and quantification of proliferation (*n* = 7) and cytokine production (*n* = 4) in CD8^+^ T cells. **h** NDV-preinfected SUDHL4 cells were co-cultured with CellTrace violet-stained PBMCs +/− Staphylococcal enterotoxin B (SEB). CD8^+^ T cells (*n* = 5) were analyzed after 3 days. **g**, **h** Two-way ANOVA with Sidak’s multiple comparisons test. Data show mean ± SD.

Given the key role of DCs in promoting durable T cell immunity we analyzed DCs and found that although NDV treatment did not affect the proportion of i.t. DCs, they accounted for only 0.1–0.5% of all infiltrating immune cells (Fig. 1k). Furthermore, DC numbers were significantly reduced in the tumor-draining lymph nodes (TdLNs) of NDV-treated mice, possibly a result of direct infection of i.t. DCs reducing migration to TdLNs (Fig. 1k). We therefore hypothesized that expanding i.t. DCs might increase cross-presentation of tumor Ag released upon NDV infection leading to an enhanced adaptive and long-term anti-tumor immune response. For this purpose, we assessed administration of the growth factor Flt3L that is critical for DC development and promotes the expansion of immature DCs in mice and humans^2,7,28^. Because DC activation is required for efficient T cell priming, we first analyzed the effects of NDV-induced tumor cell death on co-cultured splenocytes from both untreated and Flt3L-treated mice, by a 25-color spectral flow cytometry panel to quantify the activation state of different populations (Fig. 2a). viSNE analysis revealed that NDV-killed tumor cells preferentially induced activation of Lin^-^I-Ad^+^CD11c^+^ conventional DCs (cDCs), as indicated by upregulation of MHC II, co-stimulatory molecules (CD86, CD40) and PDL1 (Fig. 2a), with similar effects in both cross-presenting XCR1^+^ cDC1 and CD11b^+^ cDC2 subsets (Supplementary Fig. 2a). NDV also induced the activation of Lin^-^B220^+^Ly6c^+^ plasmacytoid DCs (pDCs), shown as upregulation of CD40 and PDL1 (Supplementary Fig. 2a), while other myeloid cell populations were mainly unresponsive. Despite significant accumulation of DCs upon in vivo Flt3L treatment (Fig. 2a), DC activation on a per cell basis was similar in untreated and Flt3L-treated splenocytes (Fig. 2a, right panel), confirming the immature phenotype of Flt3L-expanded DCs. We next tested the effect of NDV-killed tumor cells on human monocyte-derived DCs (Supplementary Fig. 2b), as well as on peripheral blood DCs from lymphoma patients treated with Flt3L, whose proportion of cDCs (as well as pDCs) is significantly increased post Flt3L (Fig. 2b and Supplementary Fig. 2c, study NCT01976585). Consistent with murine data, expression of HLA-DR, CD86, CD40 and PDL1 was significantly increased in CD141^+^ cDC1 DCs (equivalent to mouse XCR1^+^) and CD1c^+^ cDC2 DCs (equivalent to mouse CD11b^+^), but not in pDCs, both pre- and post-Flt3L, upon co-culture with NDV-preinfected tumor cells (Fig. 2b and Supplementary Fig. 2c). To identify mechanisms of DC activation we first tested the role of type I IFNs because of their documented role in DC maturation and function^29,30^. IFNAR-blockade partially reduced DC activation per reduced expression of CD86 and PDL1, but not of I-Ad, H2Kd and CD40 (Fig. 2c). Since APCs can also be activated by damage-associated molecular patterns (DAMPs) from dead cells, we assessed the expression of DAMP receptors upon exposure to NDV-infected tumor cells, and their effect on DC activation. Co-culture of DCs with NDV-infected tumor cells dramatically increased the expression of Axl, a phagocytic receptor that binds apoptotic cell phosphatidylserine through GAS6 and PROS1 proteins^31^, and Clec9A, a C-type lectin that binds dead cell debris through exposed F-actin-myosin complexes and facilitates cross-presentation of dead-cell Ag^32^ (Fig. 2d). While Clec9A-blockade did not affect DC activation, Axl-inhibition reduced MHC II, CD86, CD40 and PDL1 expression (Supplementary Fig. 2d).

The induction of both Axl and Clec9A further suggests that NDV promotes both tumor Ag uptake and cross-presentation of tumor Ag by DCs. We therefore tested the ability of these DCs to capture tumor Ag upon NDV-induced tumor cell death (using GFP as tumor Ag). While the percentage of cells taking up GFP was slightly increased by NDV in murine splenocytes, this was markedly enhanced in splenocytes from Flt3L-treated mice, consistent with an expanded pool of DCs, and Ag uptake was associated with increased MHC I expression (Fig. 2e). Within APC subsets, NDV substantially increased Ag uptake by cDC2s and even more so by cDC1s (Supplementary Fig. 2e). Importantly, similar results were obtained when DCs from Flt3L-treated patients were co-cultured with NDV-pretreated GFP^+^ SUDHL4 cells (consistent with the increased proportion of DCs post Flt3L, Fig. 2b and Supplementary Fig. 2c). Again, we observed increased MHC I expression and tumor Ag uptake from NDV-killed tumor cells and higher GFP uptake in the cDC1 subset (Fig. 2f).

To test the ability of these DCs to cross-present captured tumor Ag to CD8^+^ T cells, we generated CRISPR gene-edited β2m^−/−^ GFP^+^ A20 cells, which are unable to directly present GFP on MHC I. Splenocytes from untreated or Flt3L-treated mice were co-cultured with NDV-preinfected β2m^−/−^ GFP^+^A20 cells; JEDI T cells were then added to the co-cultures and their activation assessed (Fig. 2g, left panel). NDV-induced cell death resulted in the cross-presentation of GFP tumor Ag, per the increased anti-GFP CD8^+^ T cell proliferation and IFN-γ production; this was significantly increased with the use of Flt3L-splenocytes (Fig. 2g, right panel). CD11c-depletion and MHC I-blockade abrogated this response, ruling out non-specific T cell activation mediated by cells other than APCs or by a pro-inflammatory cytokine milieu induced by viral infection (Supplementary Fig. 3a). Furthermore, IFNAR-blockade completely abrogated T cell activation, demonstrating the importance of type I IFNs in the context of T cell cross-priming (Supplementary Fig. 3b). Since pDCs are an important source of NDV-induced IFN I^33^ we next assessed cross-presentation with pDC-depleted splenocytes. pDC-depletion reduced T cell cross-priming to levels observed in the untreated splenocytes condition (Supplementary Fig. 3b), indicating a role for pDCs in augmenting cDC-mediated cross-presentation. Lastly, no T cell activation was observed when using GFP-negative β2m^−/−^ tumor cells, indicating a tumor Ag-specific response (Supplementary Fig. 3a).

To translate these results to the human setting, we developed an assay using Staphylococcal enterotoxin B (SEB) that binds MHC II on APCs and TCR on T cells, serving as a surrogate for Ag-specific MHC/TCR interactions (Fig. 2h). CD8^+^ T cells co-cultured with NDV-preinfected tumor cells in the presence of SEB showed significantly greater proliferation and IFN-γ production than those cultured with untreated tumor cells (Fig. 2h); CD4^+^ T cells similarly increased their TNF and IFN-γ production (Supplementary Fig. 3c). These effects were not observed in the absence of SEB indicating that T cell activation following NDV-induced cell death was contact-dependent and mediated by MHC II-expressing APCs.

Collectively, these results highlight the ability of Flt3L to mobilize immature DCs and of NDV-killed tumor cells to both load these DCs with tumor Ag and activate them to potentiate cross-priming of tumor-reactive CD8^+^ T cells.

### NDV therapy in DC-enriched tumors potentiates myeloid activation, type I IFN-dependent T cell activation, and tumor regressions

**Fig. 3:**
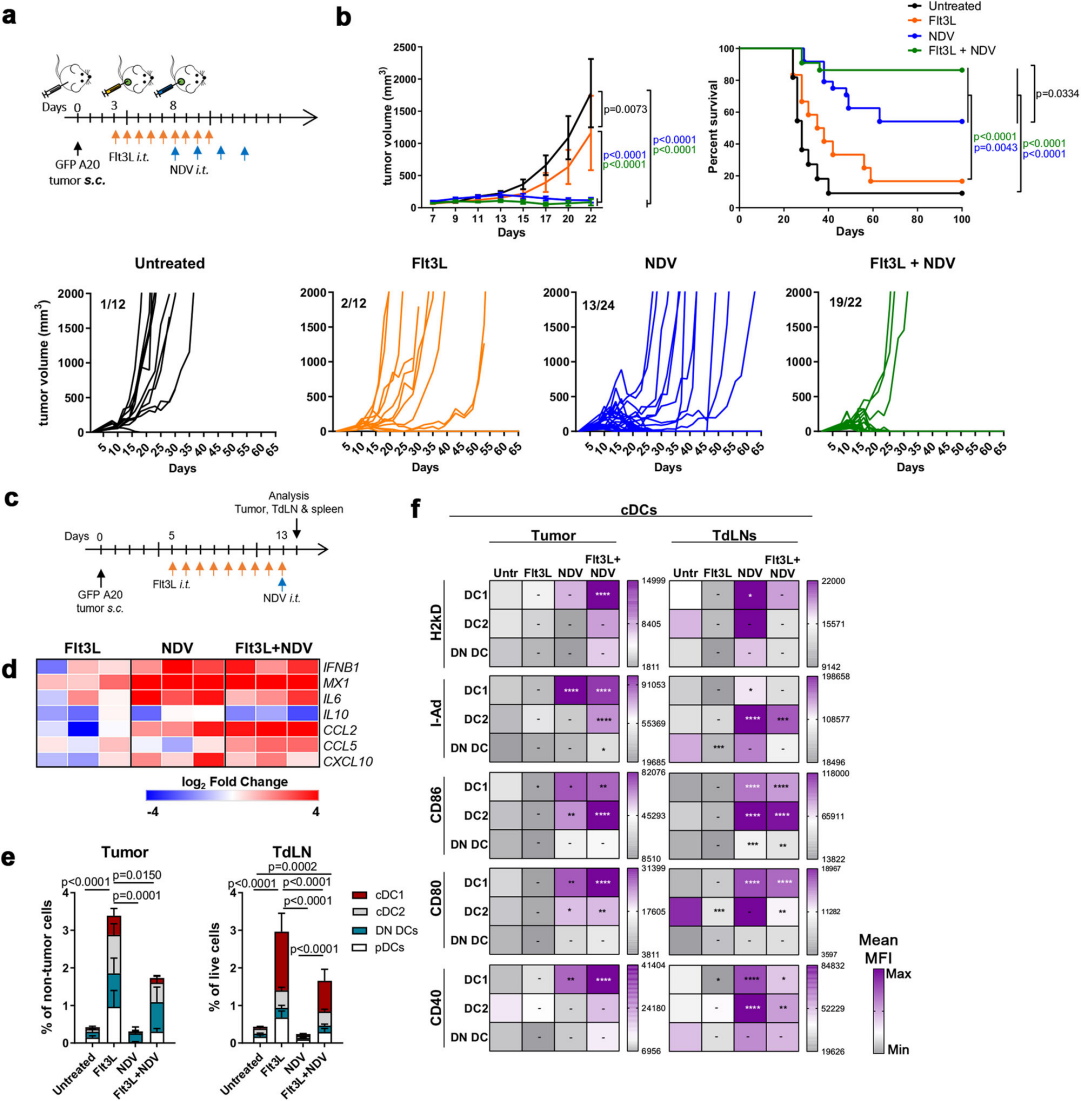
NDV therapy in DC-enriched tumors potentiates myeloid activation and tumor regressions. **a** GFP^+^ A20 tumor-bearing mice were treated as indicated and (**b**) followed for tumor growth (mean ± s.e.m, or individual growth curves, lower panel, numbers refer to mice with complete remission (CR) versus total number of mice (CR/total) in each group) and survival; data from untreated (untr, *n* = 11), Flt3L (*n* = 12), NDV (*n* = 24) and Flt3L+NDV (*n* = 22) groups, pooled from 2 independent experiments. Two-way ANOVA with Tukey’s multiple comparisons test (tumor size) and Log-rank (Mantel-Cox) test (survival). **c** GFP^+^ A20 tumor-bearing mice were treated as indicated and tumor, TdLNs and spleens were harvested and analyzed by quantitative RT-PCR (heat map, log2 fold changes vs ‘Untreated’) (**d**) or by spectral flow cytometry (**e**, **f**) (*n* = 5). **e** Stacked bar graphs of cDC1 (XCR1^+^), cDC2 (CD11b^+^), DN (XCR1-CD11b double-negative) cDC (CD11c^+^I-Ad^+^) subsets and pDCs (B220^+^Ly6C^hi^ CD11c^low^I-Ad^low^) in the tumor or TdLNs. Statistics show differences in the total DC population between treatments. One-way ANOVA with Tukey’s multiple comparisons test. **f** The heat maps show the relative expression (mean MFI of 5 individual mice) of activation markers in different cDC subsets. Two-way ANOVA with Holm-Sidak’s multiple comparisons test (vs untreated). Data show mean ± SD. *p < 0.05, **p < 0.01, ***p < 0.001, ****p < 0.0001.

The increased ex vivo T cell cross-priming by DCs with Flt3L and NDV prompted us to assess their in vivo anti-tumor immune effects. GFP^+^ A20 tumor–bearing mice received i.t. Flt3L and NDV as in Fig. 3a. While Flt3L treatment alone resulted in a small delay in tumor growth, it did not affect survival. Strikingly, combining Flt3L and NDV significantly improved long-term tumor control compared to NDV alone yielding durable remission in most mice (Fig. 3b). Furthermore, the beneficial effect of increasing DCs by Flt3L administration was even more pronounced when using a lower dose of NDV, where long-term survival was increased from 20% with NDV alone, to 70% with Flt3L+NDV (Supplementary Fig 4a).

To better understand the immunologic mechanisms of these therapies we first analyzed early effects of the treatment (24 h after a single NDV dose, Fig. 3c). Targeted tumoral gene expression analysis revealed a pro-inflammatory signature induced by NDV, with upregulation of type I IFN signaling (*IFNB1*, *MX1)* (Fig. 3d). NDV also increased expression of the IFN-inducible chemokine *CXCL10*, implicated in DC-mediated T cell recruitment^3^, and in combination with Flt3L, also *CCL5* that has been implicated in early T cell recruitment to the tumor^34^. Notably, the anti-inflammatory cytokine *IL10* was not upregulated indicating a shift toward a pro-inflammatory anti-tumor microenvironment (Fig. 3d).

**Fig. 4:**
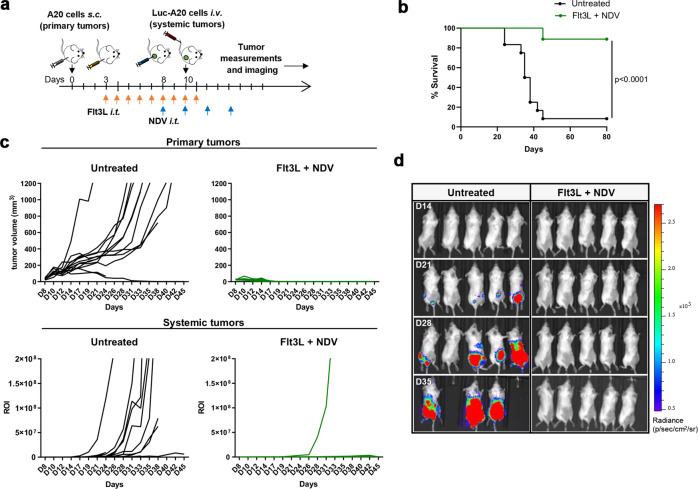
Flt3L + NDV combination therapy induces efficient systemic anti-tumor immunity. **a** GFP^+^ A20 tumor-bearing mice were treated as indicated and additionally challenged with systemic luciferase^+^ (Luc) A20 tumors. Mice were followed for survival (**b**), primary tumor growth (**c**, upper panel) and systemic tumor growth (**c**, lower panel, quantification of luminescence signal). Log-rank (Mantel-Cox) test (**b**). **d** Representative bioluminescence imaging on days 14, 21, 28 and 35 post tumor inoculation. *n* = 12 (untreated), *n* = 9 (Flt3L + NDV).

High dimensional spectral flow cytometry analysis confirmed a significant increase in DCs both in the tumor and TdLNs upon Flt3L treatment (Fig. 3e and Supplementary Fig. 4b, c), comparable to our prior data with similar approaches^7^. A small increase in Ly6c^+^ monocytes was also observed but not in other myeloid populations such as macrophages or neutrophils (Supplementary Fig. 4d). cDC1, cDC2, XCR1-CD11b double negative (DN) cDCs and pDCs all increased in the tumor, with cDC1s accounting for the largest proportion of accumulated DCs in the TdLN (Fig. 3e and Supplementary Fig. 4c). While the Flt3L-only cohort mobilized immature DCs, NDV treatment induced significant activation of tumoral and TdLN cDCs (MHC I, MHC II, CD80, CD86 and CD40) and pDCs (MHC II, CD86) (Fig. 3f and Supplementary Fig. 4e). In the tumor, this effect was most prominent in the Flt3L+NDV cohort, with significantly higher expression of activation markers (e.g. MHC I/II, CD80, CD40), particularly in cDC1s, suggesting greater-than-additive effect when combining NDV and Flt3L (Fig. 3f). This may in part be a result of the increased numbers of DCs that are susceptible to NDV infection^35^ and that may amplify the early inflammatory response, as described for other viruses^16^. Further, the effects of Flt3L and NDV on DC expansion and activation were not restricted to the tumor and TdLNs but were also observed in the spleen (Supplementary Fig. 5a) indicating a systemic effect that may benefit a systemic immune response. To test this, tumor-bearing mice treated i.t. with NDV + FLt3L (versus untreated) were additionally challenged systemically (through i.v. injections of luciferase-expressing A20 cells, Fig. 4a) and followed for tumor growth and survival. While untreated mice quickly developed tumors, the NDV + Flt3L treatment protected from both primary and systemic tumor growth, with complete regression in the majority of mice (Fig. 4b–d), clearly demonstrating that the combined treatment induces efficient systemic anti-tumor immunity. Importantly, there was no measurable toxicity associated with the combined treatment as shown by maintained body weight and no increased levels of liver and kidney enzymes after initiation of NDV treatment (Supplementary Fig. 5b, c).

**Fig. 5:**
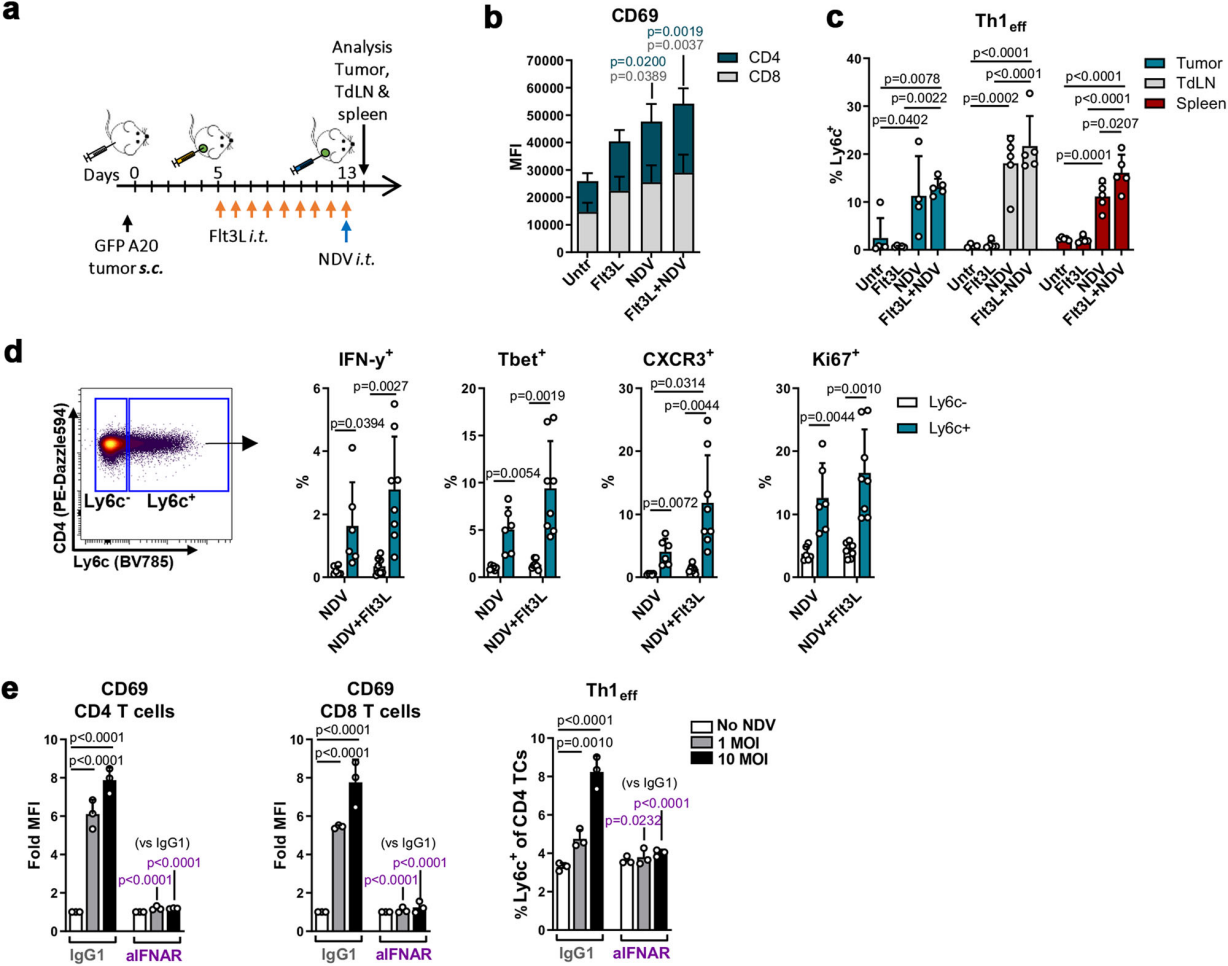
NDV therapy induces type I IFN-dependent T cell activation. **a** GFP^+^ A20 tumor-bearing mice were treated as indicated and tumor, TdLNs and spleens were harvested and analyzed by spectral flow cytometry (**b**–**d**). **b** Stacked bar graphs of activation marker expression on intratumoral CD8^+^ and CD4^+^ T cells. One-way ANOVA with Tukey’s multiple comparisons test (vs untreated). **c** Percent Ly6c^+^ Th1 effector cells (Th1_eff_) within CD4^+^ T cells. One-way ANOVA with Tukey’s multiple comparisons test; *n* = 5 mice per group (**b**, **c**). **d** Tumors from NDV-alone or Flt3L+NDV-treated GFP^+^ A20 tumor-bearing mice were harvested and IFN-γ, Tbet, CXCR3 and Ki67 were analyzed in Ly6C^+^ vs Ly6C^−^ CD4^+^ T cells. Paired, two-tailed t-test; NDV, *n* = 6; Flt3L+NDV, *n* = 8. Representative from 2 independent experiments. **e** Uninfected or NDV-preinfected A20 cells were co-cultured with splenocytes in the presence of 20 μg/ml IFNAR-blocking or isotype-control antibodies. After 24 h, T cells were analyzed by flow cytometry; Two-way ANOVA with Dunnett’s multiple comparisons test. Data show mean ± SD.

Next, we evaluated early tumor lymphocyte activation upon treatment (Fig. 5a). Both CD8^+^ and CD4^+^ T cells, as well as NK cells, were activated by NDV (CD69 and CD25 upregulation, respectively); activation was similar in NDV alone- and Flt3L+NDV-treated mice (Fig. 5b and Supplementary Fig 5d), suggesting that early lymphocyte activation is mainly DC-independent. Unsupervised viSNE analysis further revealed a striking shift in the CD4^+^ T cell population in NDV or Flt3L+NDV-treated tumors (Supplementary Fig 4b); this shift was primarily driven by Ly6c and PDL1 expression induced by NDV (Supplementary Fig 4b). Since Ly6c, a receptor regulated by Tbet^36^, defines Th1 effector cells in the context of viral infection^37^, we analyzed this more closely and found significant upregulation on T cells in tumors, TdLNs and the spleen, in both NDV alone- and Flt3L-NDV-treated mice (Fig. 5c). These Ly6c^+^ T cells expressed substantially higher levels of IFN-γ, Tbet and the CXCL9/10/11 receptor CXCR3 and proliferated to a higher extent than Ly6c^−^ CD4^+^ T cells (Fig. 5d and Supplementary Fig 5e) confirming their activated Th1 effector phenotype. To determine if type I IFN signaling was helping to drive the T cell phenotypes, we co-cultured NDV-infected A20 cells with splenocytes in the presence of an IFNAR-blocking antibody. Similar to the effect on tumor Ag-specific T cell cross-priming (Supplementary Fig 3b), activation of CD8^+^ and CD4^+^ T cells, as well as Ly6c^+^ Th1 effector cells, were completely abrogated (Fig. 5e), indicating that type I IFN signaling also is a main inducer of early T cell activation upon NDV infection. Altogether these results show that NDV triggers a broad pro-inflammatory response that is amplified by Flt3L-mobilized DCs, the combination of which induce durable tumor regressions and a significant increase in survival. Further, the modest effect of Flt3L on i.t T cell activation at this early timepoint, suggests that the impact of Flt3L on DC-mediated tumor Ag cross-presentation may play a key role in preventing late tumor relapses in the combined treatment.

### Combination therapy enhances viral and tumor Ag T cell responses in vivo

**Fig. 6:**
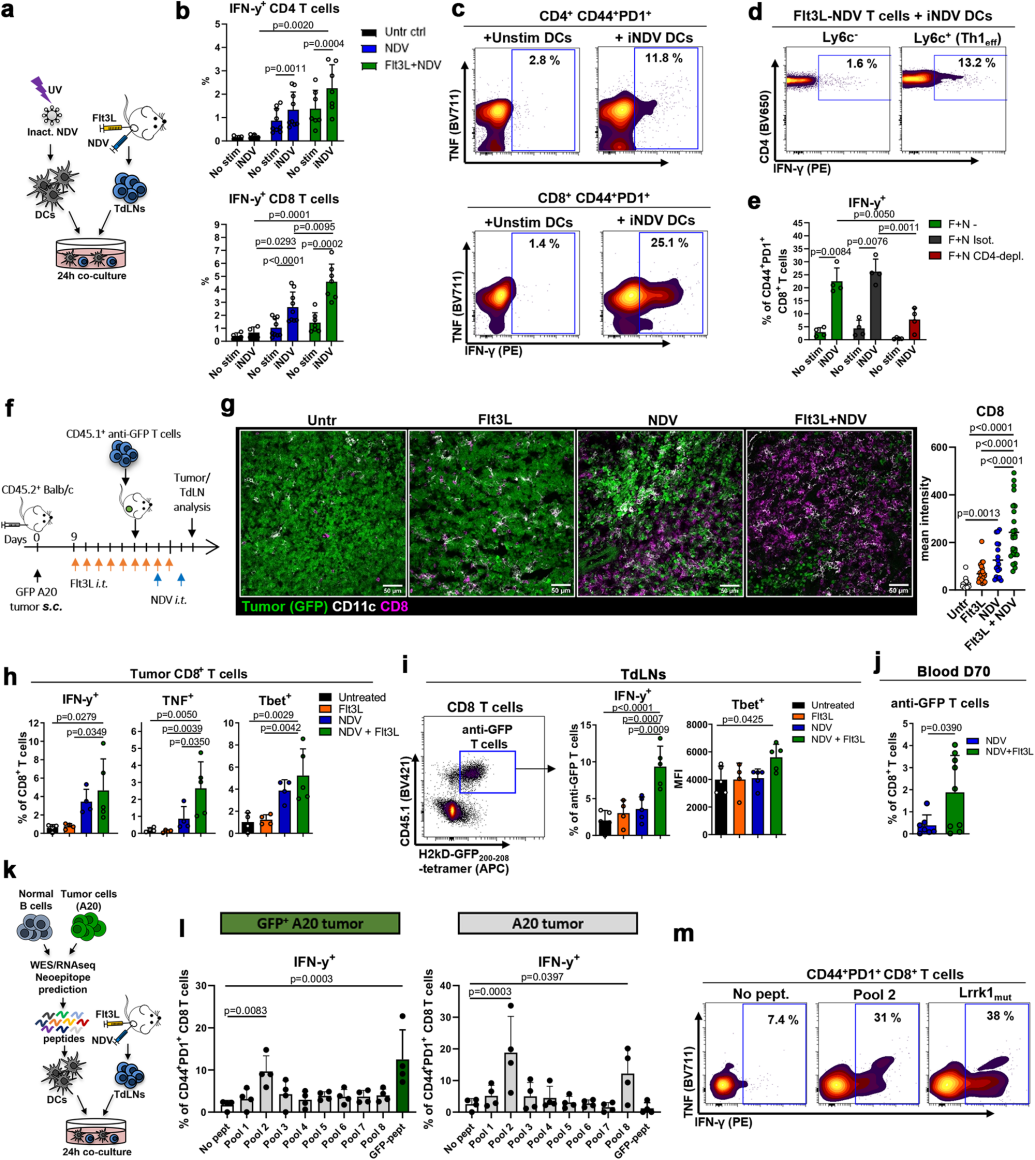
Combination therapy enhances viral and tumor Ag responses in vivo. **a**–**d** TdLNs from untreated (ctrl), NDV- or Flt3L+NDV-treated A20-tumor-bearing mice were co-cultured with DCs: unstimulated (No stim) or pulsed with UV-inactivated NDV (iNDV); T cells were analyzed after 24 h. **b** IFN-γ^+^ T cells in untreated (*n* = 4), NDV (*n* = 9) and Flt3L+NDV (*n* = 7) groups, pooled from 2 independent experiments. Paired, two-tailed t-test (No stim vs iNDV) or one-way ANOVA with Tukey’s multiple comparisons test for comparison between treatments. **c**, **d** Percentage of iNDV-reactive cells within CD44^+^PD1^+^ CD4^+^ (top) or CD8^+^ (bottom) T cells (**c**) and within Ly6C^−^ vs Ly6C^+^ CD4^+^ T cells (**d**). **e** TdLN T cells from Flt3L+NDV-treated A20-tumor-bearing mice pre-treated with CD4-depleting or isotype-control antibodies were analyzed as in (**a**). Paired, two-tailed t-test (No stim vs iNDV) or one-way ANOVA with Tukey’s multiple comparisons test for comparison between treatments (*n* = 4). **f** GFP^+^ A20-tumor-bearing mice were treated as indicated, anti-GFP CD45.1^+^CD8^+^ T cells were adoptively transferred, and tumoral*/*TdLN T cells were analyzed after 5 days by immunofluorescence (**g**) or spectral flow cytometry (**h**, **i**). **g** Representative tumor images, and CD8 mean intensity from a total of 18-27 20x images per group (Untr and NDV, *n* = 2; Flt3L and Flt3L+NDV, *n* = 3); One-way ANOVA with Tukey’s multiple comparisons test. **h** IFN-γ, TNF and Tbet expression in intratumoral CD8^+^ T cells. **i** Representative dot plot showing anti-GFP JEDI T cells in the TdLN (left) and bar graphs of JEDI IFN-γ and Tbet expression (right). **h**, **i** *n* = 5 mice per group; one-way ANOVA with Dunnett’s multiple comparisons test. **j** Mice with complete remissions (NDV, *n* = 7; Flt3L+NDV, *n* = 9) were analyzed for blood tetramer^+^ anti-GFP CD8^+^ T cells day 70 after tumor inoculation. Unpaired, two-tailed t-test. **k**–**m** TdLN cells from Flt3L+NDV-treated A20 or GFP^+^ A20-tumor-bearing mice were co-cultured with DCs pulsed with pooled or individual neoepitope peptides identified by exome and RNA sequencing, or GFP-peptide. **l**, **m** IFN-γ production after 24 h; data pooled from 2 independent experiments, *n* = 4 mice per tumor type, one-way ANOVA with Dunnett’s multiple comparisons test (**l**) and representative (*n* = 4) contour plots of CD44^+^PD1^+^CD8^+^ T cells reactive to peptide pool 2 and Lrrk1_mut_ (**m**). Data show mean ± SD.

Next, we sought to determine the specificity of the immune response induced by NDV and Flt3L. Because CD8^+^ T cells were found in close association with NDV-infected tumor cells (Fig. 1h), we first assessed for NDV-reactive T cells. We developed an assay using splenic DCs pulsed with UV-inactivated NDV (iNDV) (Supplementary Fig. 6a) and co-cultured these with TdLN cells from tumor-bearing mice (Fig. 6a). iNDV-reactive IFN-γ-producing cells were found amongst both CD4^+^ and CD8^+^ T cell populations in TdLNs from NDV-treated mice, with significantly more IFN-γ-producing CD8^+^ T cells in Flt3L+NDV-treated mice (accounting for up to 25% of Ag-experienced CD44^+^PD1^+^ cells) versus control mice (Fig. 6b, c and Supplementary Fig. 6b). In CD4^+^ T cells, IFN-γ-producing cells were predominantly found in the Ly6c^+^ population (Fig. 6d), consistent with NDV treatment inducing an anti-viral Th1 effector response. To assess whether these virus-reactive CD4^+^ T cells were functionally helping CD8^+^ T cell responses, we performed this assay with TdLNs from in vivo CD4-depleted mice. We observed a marked reduction in IFN-γ-producing CD44^+^PD1^+^ CD8^+^ T cells (Fig. 6e), suggesting that activated CD4^+^ T cells help amplify the anti-viral CD8^+^ T cell response.

Next, we asked whether the combined Flt3L and NDV treatment resulted in enhanced tumor-specific T cell activation, in particular the cross-priming of CD8^+^ T cells that are critical for efficient tumor control. We first assessed the tumor-specific response by adoptive transfer of naïve anti-GFP JEDI T cells into GFP^+^A20-bearing mice prior to treatment (Fig. 6f). Immunofluorescence demonstrated significant CD8^+^ T cell infiltration into NDV-treated tumors, but a dramatic additional increase in the Flt3L+NDV group (9-fold greater than in untreated mice) (Fig. 6g). Spectral flow cytometric analysis further showed highest CD25 and CD69 expression in CD8^+^ T cells (as well as CD4^+^ T cells) in Flt3L+NDV-treated mice (Supplementary Fig. 6c), similar to the results seen with endogenous T cells in the absence of JEDI T cells (Fig. 5b). Furthermore, significantly more IFN-γ^+^, TNF^+^ and Tbet^+^ CD8^+^ T cells were observed in tumors treated with both NDV and Flt3L (Fig. 6h), consistent with the greater-than-additive effect of combined Flt3L+NDV treatment on DCs (Fig. 3f). Along with endogenous CD8^+^ T cells (CD45.1^−^), JEDI CD8^+^ T cells (CD45.1^+^) were also present in the tumor and showed a similar pattern of activation and consistently higher expression of IFN-γ, TNF and Tbet than endogenous CD8^+^ T cells (Supplementary Fig. 6d), implying tumor Ag-specific cross-priming by cDC1s and T cell recruitment to the tumor. Accordingly, IFN-γ^+^ and Tbet^+^ JEDI T cells were significantly more abundant in the TdLNs of Flt3L+NDV-treated than untreated, Flt3L- or NDV-only treated mice (Fig. 6i). Additionally, we observed –in addition to the adoptively transferred anti-GFP JEDI T cells– a significant population of endogenous, i.t. anti-GFP CD8^+^ T cells per their IFN-γ and Tbet expression upon ex vivo GFP-peptide stimulation, demonstrating that Flt3L+NDV therapy induces tumor Ag-specific T cell responses (Supplementary Fig. 6e).

T cell activation is associated with induction of immune inhibitory receptors that limit the anti-tumor response. Although a tendency towards increased expression of the inhibitory PD1, Tim3 and Lag3 receptors was observed on i.t. CD8^+^ T cells in Flt3L+NDV-treated compared with untreated, Flt3L- or NDV-treated tumors, it did not reach statistical significance (Supplementary Fig. 6f). To test whether the specific anti-tumor immune response was maintained long-term and in the absence of adoptively transferred T cells, mice with complete remissions in Fig. 3b were re-challenged with GFP^+^ A20 tumors in the opposite flank 60 days after completed NDV or NDV-Flt3L treatment. While all mice were protected from tumor re-growth, a significantly higher proportion of anti-GFP CD8^+^ T cells was found in the blood of Flt3L+NDV-treated than NDV-only treated mice (Fig. 6j). These results indicate that mobilizing i.t. DCs in the context of NDV therapy is beneficial for the maintenance of tumor-specific T cell memory.

### Flt3L and NDV generates neoepitope-specific CD8^+^ T cells

Induction of T cell responses against a model Ag such as GFP demonstrates that this approach can increase tumor Ag cross-presentation. Still, the anti-GFP T cells induced by Flt3L and NDV comprised only a minority of CD8^+^ T cells in the tumor and TdLN (Supplementary Fig. 7a). Translationally relevant therapies should induce T cells specific for endogenous -and less immunogenic- tumor Ag. To date, no neoepitope-reactive T cell responses have been demonstrated by NDV-based therapies, or for murine lymphomas. We therefore performed whole-exome and RNA sequencing to identify expressed A20 somatic mutations and synthesized 82 unique 14-25-mer peptides containing predicted immunogenic neoepitopes (Supplementary Table 1). TdLN cells from Flt3L+NDV-treated A20- or GFP^+^ A20-bearing mice were co-cultured with DCs pulsed with candidate neoepitope peptides (initially pooled into groups of 10-11 peptides) and T cells were analyzed for IFN-γ production (Fig. 6k). A large proportion of CD8^+^ T cells from both A20- or GFP^+^ A20-bearing mice produced IFN-γ in response to peptide pool 2, in the latter to the same extent as in response to GFP-peptide (~10% of CD44^+^PD1^+^ CD8^+^ T cells and 0.5% of all CD8^+^ T cells, Fig. 6l and Supplementary Fig. 7b). Although CD8^+^ T cells also produced IFN-γ in response to peptide pool 8, this was only observed in A20 tumor-derived T cells (Fig. 6l). Further analysis of individual peptides showed that CD8^+^ T cells predominantly reacted to a mutated form of Leucine-rich repeat kinase 1 (Lrrk1_mut_) (peptide 18, Fig. 6m and Supplementary Fig. 7c), and that this reactivity was restricted only to short sequences containing the mutated amino acid (Supplementary Fig. 7d). Similar to anti-viral and anti-GFP responses, the Flt3L+NDV combined treatment elicited a stronger neoepitope response than NDV-alone treatment (Supplementary Fig. 7e). These results are a proof-of-principle that augmenting DCs in the context of oncolytic therapy achieves neoepitope immune responses without resource- and time-intense personalized treatment strategies.

### Batf3-DCs are critical for the anti-tumor effects of Flt3L + NDV

**Fig. 7:**
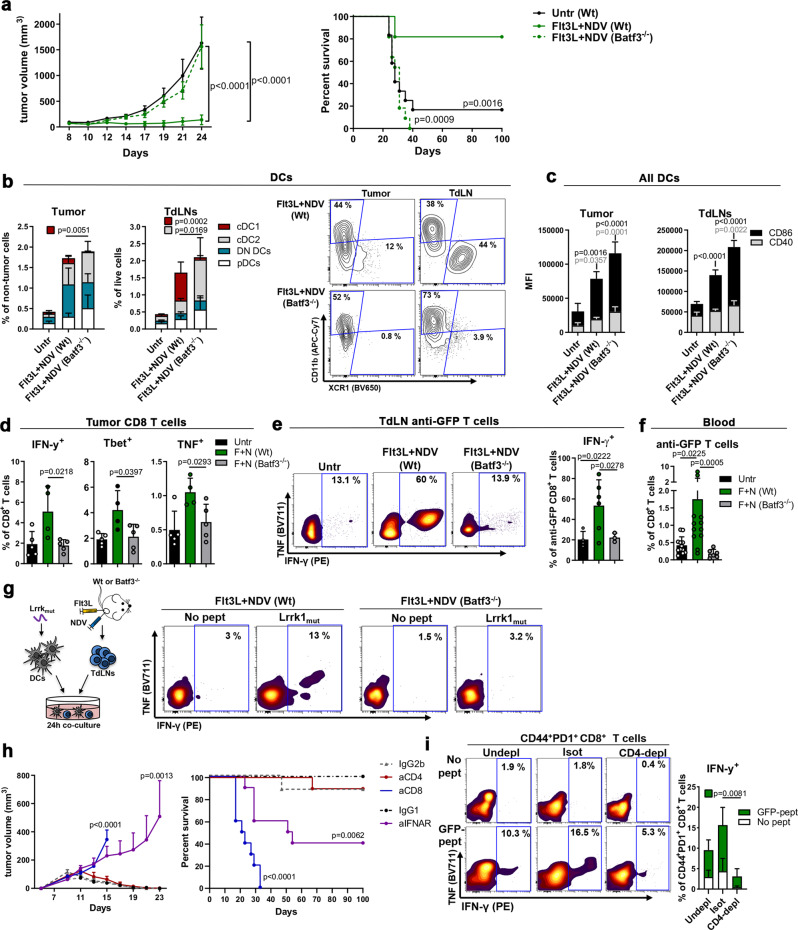
Batf3-DCs are critical for the anti-tumor effects of Flt3L + NDV. **a** Flt3L+NDV-treated GFP^+^ A20-tumor-bearing Wt or Batf3^−/−^ mice were followed for tumor growth (mean ± s.e.m) and survival (untreated, *n* = 12; Flt3L+NDV Wt, *n* = 11; Flt3L+NDV Batf3^−/−^, *n* = 11). Two-way ANOVA with Tukey’s multiple comparisons test (tumor size) and Log-rank (Mantel-Cox) test (survival). **b**, **c** Tumors/TdLNs from treated GFP^+^ A20-tumor-bearing mice were analyzed by spectral flow cytometry. Stacked bar graphs and representative contour plots of cDC1, cDC2, DN cDCs or pDCs (**b**) and stacked bar graphs of CD86/CD40 expression (vs Untr) in all cDCs (**c**). **b**, **c** One-way ANOVA with Tukey’s multiple comparisons test; *n* = 5 mice per group. **d**, **e** GFP^+^ A20-tumor-bearing Wt or Batf3^−/−^ mice were treated with Flt3L+NDV. Anti-GFP CD45.1^+^CD8^+^ (JEDI) T cells were adoptively transferred and tumoral/TdLN T cells were analyzed after 5 days. IFN-γ, TNF and Tbet expression in intratumoral CD8^+^ T cells (Untr; *n* = 5, Flt3L+NDV Wt, *n* = 4, Flt3L+NDV Batf3^−/−^, *n* = 5) (**d**) and representative contour plots and bar graphs showing IFN-γ in JEDI T cells from TdLNs (after completed treatment) upon GFP-peptide stimulation ex vivo (**e**) (Untr; n = 4, Flt3L+NDV Wt, *n* = 6, Flt3L+NDV Batf3^−/−^, *n* = 3). **f** Mice in (**a**) were analyzed for blood anti-GFP tetramer^+^CD8^+^ T cells 7 days after completed treatment. Kruskal-Wallis with Dunn’s multiple comparisons test; *n* = 12 (untr, Flt3L+NDV Wt) and *n* = 6 (Flt3L + NDV Batf3^−/−^). **g** TdLN cells from tumor-bearing Flt3L+NDV-treated mice were co-cultured with Lrrk1_mut_ peptide-pulsed DCs; T cells were analyzed after 24 h. IFN-γ in CD44^+^PD1^+^CD8^+^ T cells, representative from two A20- and three GFP^+^ A20-bearing mice. **h** GFP^+^ A20-tumor-bearing Wt mice treated with anti-CD8, anti-CD4, anti-IFNAR or isotype-control antibodies were treated with Flt3L+NDV and followed for tumor growth (mean ± s.e.m) and survival. Two-way ANOVA with Sidak’s multiple comparisons test (tumor size) and Log-rank (Mantel-Cox) test (survival); *n* = 8 (IgG2b), *n* = 9 (IgG1, anti-CD8), *n* = 10 (anti-CD4, anti-IFNAR). **i** TdLNs from mice treated as in (**g**) with anti-CD4 or isotype-control antibodies and Flt3L+NDV were co-cultured with unstimulated (No pept) or GFP-peptide pulsed (GFP-pept) DCs and analyzed after 24 h. Representative contour plots and quantification of IFN-γ in CD44^+^PD1^+^CD8^+^ T cells. One-way ANOVA with Tukey’s multiple comparisons test, *n* = 4 mice per group. Data show mean ± SD.

To determine the role of cross-presenting cDC1s in the efficacy of the Flt3L+NDV treatment, GFP^+^ A20 tumor-bearing Wt or Batf3^-/-^ mice were treated as previously described (Fig. 3a). The therapeutic effect of Flt3L+NDV was completely lost in Batf3^−/−^ mice (Fig. 7a), despite similar increase and activation of total cDCs and pDCs in the tumor and TdLN upon Flt3L+NDV treatment (Fig. 7b, c and Supplementary Fig 8a); an increased proportion of cDC2s was observed, possibly to compensate for the lack of cDC1s (Fig. 7b). Detailed analysis of the tumor microenvironment showed that the early effects on immune cell activation were not dependent on cross-presenting cDC1s since Batf3^−/−^ mice showed similar or higher expression of early activation markers in CD8^+^ T cells (CD25, CD69), CD4^+^ T cells (CD25, CD69, Ly6c) and NK cells (CD25) (Supplementary Fig 8b, c). However, returning to the JEDI T cell adoptive transfer model (Fig. 6f), the percentage of i.t. IFN-γ^+^, TNF^+^ and Tbet^+^ CD8^+^ T cells in Flt3L+NDV-treated Batf3^−/−^ mice was reduced to levels seen in untreated mice (Fig. 7d). Similarly, JEDI CD8^+^ T cells derived from the TdLN of Batf3^−/−^ mice showed deficient production of IFN-γ in response to ex vivo GFP-peptide stimulation (Fig. 7e), and the induction of circulating anti-GFP T cells observed in Wt mice was also lost in Batf3^−/−^ mice (Fig. 7f). As with anti-GFP responses, the induction of neoepitope-reactive CD8^+^ T cells in the TdLN was completely lost in Batf3^−/−^ mice (Fig. 7g). By contrast, virus-reactive CD8^+^ and CD4^+^ T cells were only moderately reduced in Batf3^−/−^ mice (Supplementary Fig 8d), indicating that cDC1s are dispensable for the induction of virus-specific T cells, and that other APC subsets including cDC2s mediate early T cell priming by directly presenting viral Ag to CD4^+^ and CD8^+^ T cells, or by cross-presenting soluble Ag to CD8^+^ T cells^4,38^. These data highlight that ‘turning cold tumors hot’, with marked increases in activated DCs and T cells, including virus-specific T cells, is insufficient without cDC1s to cross-present cell-associated tumor Ag, i.e. ‘hot is not enough’.

As expected, depletion of CD8^+^ T cells (Supplementary Fig. 8e) completely abrogated the effect on tumor growth and survival with Flt3L+NDV treatment (Fig. 7h), confirming the importance of CD8^+^ T cells in anti-tumor immunity. Further, i.t. IFNAR-blockade at the time of NDV injection (Supplementary Fig. 8e) significantly reduced the effects of Flt3L+NDV on tumor growth and survival (Fig. 7h), despite similar numbers of circulating anti-GFP CD44^+^PD1^+^ CD8^+^ T cells (Supplementary Fig. 8f), suggesting that IFN I signaling plays a complementary role to cDC1-mediated anti-tumor T cell priming.

Based on the significant Flt3L-induced increase in cDC2s, that specialize in CD4^+^ T cell priming, and the striking effects of NDV on CD4^+^ Th1 effector cells, we sought to evaluate the role of CD4^+^ T cells for Flt3L+NDV-induced anti-tumor immunity in vivo. CD4 depletion (as shown in Supplementary Fig. 8e) did not affect tumor growth and survival induced by Flt3L+NDV treatment (Fig. 7h); however, it should be noted that CD4-specific antibodies also deplete tumor-promoting regulatory T cells, potentially masking the effects mediated by CD4^+^ effector T cells. Nonetheless, we found that CD4 depletion reduced the induction of circulating anti-GFP CD44^+^PD1^+^ CD8^+^ T cells (Supplementary Fig. 8f) and the ability of TdLN-derived CD44^+^PD1^+^ CD8^+^ T cells to produce IFN-γ upon GFP-peptide re-stimulation ex vivo (Fig. 7i). These results suggest that CD4^+^ T cells improve oncolytic virus-induced anti-tumor immunity by amplifying tumor-specific CD8^+^ T cell activation and could be beneficial in the context of suboptimal induction of anti-tumor CD8^+^ T cells, despite being dispensable in this setting of potently primed CD8^+^ T cells.

## Discussion

Here we demonstrate the benefit of increasing i.t. DCs for the anti-tumor effects of in situ vaccination with the oncolytic virus NDV. Using murine and patient-derived DCs we show that NDV efficiently induces cDC1 activation and tumor Ag cross-presentation, and that Flt3L induces robust amplification of tumor-specific T cell responses and long-term tumor control. Flt3L-NDV ISV therapy efficiently induced CD8^+^ T cells reactive to neoepitopes identified by tumor exome and RNA sequencing. We further show that cross-presenting cDC1s are indispensable for a functional anti-tumor CD8^+^ T cell response and tumor clearance, but also NDV-induced type I IFNs and CD4^+^ T cells play complementary roles in promoting optimal anti-tumor immunity.

CD8^+^ T cells are critical for oncolytic-virus mediated tumor rejection^17,39,40^, thus, the field has focused on boosting T cell activation by incorporating stimulatory cytokines (e.g. IL-2, IL-7, IL-12 or IFN-γ) into oncolytic vectors^17,41,42^, though such approaches have yielded only moderate benefit. While direct T cell activation may promote robust amplification of pre-existing i.t. T cells, a majority of i.t. T cells are not tumor-reactive^43^ and their activation is unlikely to facilitate anti-tumor immunity. By contrast, Batf3-DCs are indispensable for cross-priming of CD8^+^ T cells and generating de novo anti-tumor T cell responses, therefore, expanding intratumoral DCs is a rational alternative approach to augment oncolytic virotherapy. GM-CSF, which expands many myeloid cell lineages, has been broadly tested with oncolytic vectors, including the GM-CSF-expressing HSV vector T-vec, the only FDA-approved oncolytic virus to date. Still, the durable response rate is a modest 16%^44^, illustrating the limited induction of systemic anti-tumor immunity. Studies of virus-mediated DC activation have shown induction of anti-tumor CD8^+^ T cells in murine models^16,45^ but overlook the importance of first increasing the rare, but critical cDC1 subset. Whereas GM-CSF is insufficient to expand optimal cross-presenting DCs^46,47^, Flt3L uniquely promotes the expansion of all DC subsets, including cross-presenting cDC1s^48,49^. Early Flt3L clinical trials failed to induce anti-tumor immunity^50,51^, likely attributable to the tolerogenicity of immature DCs^52^; however, recent trials show that only the combination of Flt3L and DC activators (e.g. TLRa) yield potent Ag-specific T cell responses^53^. Further, while oncolytic viruses have been postulated to promote tumor Ag cross-presentation by DCs, there is a lack of mechanistic evidence explicitly demonstrating this. Our approach using JEDI T cells and visualizable Ag (GFP) demonstrates the ability of NDV to induce DC uptake of tumor-associated Ag and activation, by both murine and patient cDC1s. Interestingly, NDV upregulated DC expression of dead-cell receptors, including Clec9A that mediates cross-presentation of dead-cell Ag^32^, suggesting that NDV sensitizes DCs to dying tumor cells. Accordingly, we show that NDV stimulates MHC I-dependent CD8^+^ T cell cross-priming upon tumor cell death, amplified by Flt3L, demonstrating the benefit of cDC1 expansion. Although studies using Flt3L-expressing viral vectors have previously shown survival benefit in mice, others have shown limited effects, likely attributable to failure to induce sufficient and prolonged i.t Flt3L expression^54–56^ highlighting that timing of DC recruitment and activation relative to tumor cell death and Ag uptake is an important variable^57,58^. Importantly, combining NDV with recombinant Flt3L yielded greater-than-additive induction of durable anti-tumor T cell responses in vivo, suggesting this approach may be optimal to cross-prime anti-tumor T cells. Batf3 expression in T cells was recently shown to promote memory formation^59^; however, in that prior work, T cell defects were most apparent during contraction and in long-term memory, while neither the primary response nor the function (i.e., TNF/IFN-γ production) of CD8^+^ T cells were affected in Batf3-deficient T cells. In contrast, our studies demonstrated early defects in anti-tumor effects (Fig. 7a) and anti-tumor IFN-γ production in tumor, TdLNs, and blood of CD8^+^ T cells in Batf3-deficient animals, even when studying anti-GFP (Batf3-wt) transferred T cells (Fig. 7d–f). Therefore, it is not likely that the impaired induction of anti-tumor CD8^+^ T cells observed in Batf3^−/−^ mice in our study is caused by T cell-intrinsic Batf3-deficiency, but rather the lack of cDC1-mediated cross-priming.

Given the critical role of neoepitopes as immunotherapy targets^60,61^ it is important to determine if ‘off-the-shelf’ vaccines can elicit neoepitope-reactive T cell responses. Since immunogenic neoepitopes have not been described for murine lymphoma, we first identified potential MHC I neoepitopes and screened for reactive T cells in ISV-treated mice, and identified T cell responses to mutated Lrrk1, an NFκB driving phosphokinase^62^ frequently mutated in lymphoma and solid malignancies (cbioportal.org). NDV-Flt3L treatment elicited robust CD8^+^ T cell activation towards Lrrk1_mut_, remarkably, to a similar magnitude as the highly immunogenic xeno-antigen GFP. To our knowledge this is the first study to show induction of neoepitope-specific T cell responses by an NDV-based therapy or in lymphomas. Importantly, this is a proof-of-concept that neoepitope-reactive T cells can be induced without the resource- and time-consuming process associated with personalized neoepitope vaccines.

Oncolytic virus-specific T cells have been reported^17^, but it is unclear as to what extent they contribute to tumor clearance during oncolytic virotherapy. Our data revealed the early induction of NDV-reactive T cells, including IFN-γ-producing Ly6c^+^CD4^+^ T cells. Ly6c^+^ Th1 cells have been shown to represent terminally differentiated effector cells with high cytotoxic potential^37,63^, suggesting they could help eliminate infected tumor cells. However, while virus-reactive T cells were mostly maintained in Batf3^−/−^ mice, treatment completely failed, demonstrating the importance of tumor-specific CD8^+^ T cells for NDV-induced tumor control. We therefore suggest that although virus-specific effector T cells could help improve durable anti-tumor immunity in the context of cDC1-induced CD8^+^ T cell priming, these T cells alone do not substantially promote tumor clearance. Further, since cDC2-primed CD4^+^ T cells can improve the magnitude and quality of CD8^+^ T cells^64^, the increase in activated cDC2s upon NDV-Flt3L ISV could also benefit anti-tumor immunity. Indeed, CD4-depletion reduced induction of both virus- and tumor-reactive CD8^+^ T cells upon NDV-Flt3L treatment. Thus, although CD4-depletion did not hamper tumor rejection in our model and those of others^7,17^, our data shows that cDC2s may play an important indirect role in promoting CD8^+^ T cell memory and durable anti-tumor responses, as seen pre-clinically^65^ and in immunotherapy-treated patients^66^.

We believe that this strategy could be rapidly translated to the clinic given the completed trials showing safety of i.t. NDV, the pre-clinical superior efficacy of i.t. versus systemic NDV administration^18^, and the ongoing multi-center trials of NDV encoding genes for GM-CSF (NCT03889275) or IL-12 (NCT04613492), with over 250 patients, demonstrating clear enthusiasm. Still, there is unmet need to optimize NDV-mediated induction of durable anti-tumor immune responses, and Flt3L-mobilized DCs more effectively present antigen compared to GM-CSF-mobilized DCs^67^. Conversely, while rhFlt3L has been used in more than 10 early phase trials, poor pharmacokinetics have slowed its development. Fortunately, the newer generation of easier-to-use formulations e.g. Flt3L-Fc fusion proteins (NCT04747470) will now accelerate the growth of this field. Overall, these results demonstrate that augmenting cross-presenting DCs in the context of oncolytic therapy is an effective strategy to achieve efficient and long-term anti-tumor immunity, including the induction of neoepitope responses otherwise attributed to resource- and time-intense personalized treatment strategies.

## Methods

### Ethical compliance

Protocols for the treatment of patients, and human sample collection and analysis, were approved by the Mount Sinai Institutional Review Board, and written informed consent was obtained from all patients in accordance with the Declaration of Helsinki. All experiments including human specimens were performed in compliance with the relevant ethical regulations.

### Animals

Balb/c Wt (#000651) and Balb/c-Batf3^−/−^ (#013756) mice (8–12 weeks old) were purchased from The Jackson Laboratory. JEDI mice^25^ were provided by Dr. Brian Brown (Mount Sinai) and back-crossed on the Balb/c background for 8 generations in our facility. Both male and female mice were used in short-term in vivo and in vitro experiments, and female mice were used in experiments monitoring long-term survival. Mice used in experiments were co-housed under standard special pathogen free condition (standard 12-light/12-dark cycle, temperatures between 68–75° F and 30–70% humidity) at the animal facility of the Icahn School of Medicine at Mount Sinai. All experiments were reviewed and approved by the Institutional Animal Care and Use Committee of the Icahn School of Medicine at Mount Sinai. For tumor-challenge experiments, all animals were monitored daily by Researchers and three times per week by Facility Veterinarians or Veterinary Technicians and euthanized within 24 h if they exhibited any sign of decreased body condition (body score <2), e.g., hunched posture, sluggish movements, exceeded maximal tumor size or developed tumoral ulceration, as per IACUC-approved protocol. Animals were euthanized per the IACUC-approved procedure of 70% CO2 chamber.

### Cell lines

All cell lines were maintained at 37 °C with 5% CO_2_ and cultured in RPMI (A20) or IMDM (SUDHL4) with 10% heat-inactivated FCS, penicillin/streptomycin and 50 uM β-mercaptoethanol (A20). GFP^+^ and mCherry^+^ A20 cells were generated by transducing A20 cells with a lentivirus encoding eGFP or mCherry, and B2m^−/−^ and GFP^+^ B2m^−/−^ A20 cells were generated using CRISPR/Cas9, as previously described^7^. GFP^+^ A20 cells were passaged 5 times in Balb/c mice to generate GFP^+^ A20 cells that were able to grow in vivo with minimal rejection by naturally occurring anti-GFP T cells; these were used in all in vivo experiments with GFP^+^ A20 cells. For systemic tumor experiments, mCherry^+^ A20 cells were stably transduced with a lentiviral vector encoding firefly luciferase (Luc) and selecting for puromycin resistance. Selected mCherry^+^Luc^+^ tumor cells were passaged 3 times in Balb/c mice after which tumors were excised and digested to single cell suspension and purified based on mCherry expression by FACS sorting. mCherry and luciferase expression was confirmed by flow cytometry and IVIS Spectrum imaging, respectively. GFP^+^ SUDHL4 cells were a gift from Dr. David Dominguez-Sola (Mount Sinai).

### Oncolytic vector

Modified NDV LaSota-L289A^68^ is a lentogenic -avirulent- non-lytic viral vector with highly restricted capacity for multicycle of replication^18^. Virus stock was propagated in 9 day old embryonated chicken eggs. Titer of clear-purified virus was calculated by indirect immunofluorescence on Vero cells (African green monkey kidney epithelial cells; ATCC) using polyclonal serum to NDV^69^.

### Tumor inoculation and treatments

2.5 × 10^6^ A20 or GFP^+^ A20 cells were injected in 100 μl HBSS subcutaneously on the flank of the right hind leg. For tumor-re-challenge, 2.5 × 10^6^ cells were injected on the contralateral leg on day 60 post tumor inoculation. Tumor size was determined by caliper measurements on the indicated days (length × width × height), and mice were monitored up to 100 days post tumor inoculation. For tumor growth and survival experiments, mice were injected with recombinant human Flt3L (30 μg in 30 μl; Celldex) i.t. for 9 daily injections. Starting day 6 of FLt3L injection, mice received a total of 4 (or 2 where indicated) i.t injections of NDV (10^7^ PFU in 50 μl) every 2 days. Where indicated, cohorts of mice were injected i.t. with 500 μg/ml anti-mouse IFNAR-blocking antibodies (clone MAR1-5A3; BioXCell) or IgG1 isotype control (clone MOPC-21; BioXCell) on days −1 and 0 of the start of NDV treatment, and with 250 μg/ml anti-IFNAR or isotype control antibodies on days 2, 4, 6 and 8 post-NDV treatment. For depletion of immune cell subsets, mice were injected intraperitoneally with 200 μg anti-CD8 (2.43; BioXCell), anti-CD4 (GK1.5; BioXCell), or the respective isotype antibodies (BioXCell) four times 2 days apart, starting 2 days before start of Flt3L treatment and every 7 days thereafter (See also Supplementary Fig. 8e). For systemic tumors, 2 × 10^5^ Luc^+^ A20 cells in 200 μl HBSS were injected tail vein injection on day 10 post s.c. tumor inoculation. In vivo bioluminescence imaging was performed using an IVIS Spectrum system (PerkinElmer, purchased with the support of NCRR S10-RR026561-01) to quantify systemic tumor burden. Mice were imaged 5 min after retroorbital injection with 100 µl D-luciferin (PerkinElmer) with 1 min exposure. Luciferase signal was quantified using Living Image software (PerkinElmer). Safety of the treatment was monitored by body weight and serum levels of liver (AST, ALP, ALT) and kidney (creatinine) enzymes (IDEXX BioAnalytics) at the indicated time points.

### In vitro NDV infections

A20 or SUDHL4 lymphoma cells plated in 24-well plates (at a density of 250,000 to 10^6^ cells per well) were infected with a viral suspension of NDV at an MOI of 1 or 10 in Opti-MEM (Gibco) for 1 h, after which complete media was added. Infections were maintained for 8 h for mRNA analyses and 24 h, 48 h or 72 h for infectivity, viability and cell-surface marker expression analyses. Cryopreserved patient peripheral blood mononuclear cells (PBMCs) or lymph node biopsy samples were quickly thawed and allowed to rest overnight after which a Dead cell removal kit (Miltenyi Biotech) was used to improve viability. Samples were then infected with NDV as described above (at a density of 10^6^ cells per well) and cultured for 24 h, 48 h or 72 h (as indicated) before analysis. For co-culture experiments, the virus was removed by centrifugation after the initial 1 h incubation and cells were resuspended in complete media before further incubations.

### Single cell preparations

To obtain single cell suspensions, tumors, spleens, and lymph nodes were dissected and homogenized by forcing the tissue through a 70 μm nylon mesh with the plunger from a sterile syringe. Cell suspensions were pelleted at 500 g for 5 min at 4 °C and resuspended in Pharm Lyse lysing buffer (BD Biosciences) for 5 min to remove red blood cells. Tumor cell suspensions were depleted of tumor cells by magnetic separation using CD19 nanobeads according to the manufacturer’s protocol (Mojosort, BioLegend). Cells were stored at 4 °C until further usage.

### Conventional and spectral flow cytometry and cell sorting

Viability staining was performed in HBSS using fixable viability stain 780 (FVS780, BD Biosciences) at 1:1000 for 5 min at room temperature (RT) or 7AAD (BioLegend) according to the manufacturer’s protocol. Mouse and human surface staining was performed in FACS buffer containing 2 mM EDTA, or blocking buffer (made in house) for myeloid cell panels, using monoclonal antibodies listed in Supplementary Table 2. Mouse and human surface antibodies were used at a dilution of 1:400 and 1:200, respectively, and samples were incubated with antibodies for 15 min at RT in the dark. For intracellular staining, surface-stained cells were fixed and permeabilized using commercial buffer sets (Invitrogen), then stained with mouse or human antibodies (Supplementary Table 2) at a 1:200 or 1:100 dilution, respectively, for 30 min at 4 °C. For NDV staining, after intracellular staining with anti-NDV rabbit polyclonal serum, samples were stained with a secondary anti-rabbit antibody for 15 min at RT in the dark. For detection of GFP-specific T cells, we used H-2K^d^-HYLSTQSAL (H2K^d^-GFP_200–208_) tetramer reagent produced by the NIH Tetramer Core Facility. Samples were acquired using an LSR-Fortessa (with the FACS Diva Software, BD Biosciences), Attune (with the NxT Software, ThermoFisher Scientific) or Aurora (SpectroFlo Software Cytek) and data was analyzed with Cytobank. Cell sorting was performed on a FACSAria (BD Biosciences). For cytokine analyses, single cell suspensions were plated, unstimulated or stimulated with 1 μg/ml GFP-peptide were indicated and treated with Brefeldin A (Invitrogen) for 6 h prior to staining. Example gating strategies are shown in Supplementary Fig. 9.

### Immunofluorescence

Whole tumors and TdLNs were washed in PBS and incubated in PLP buffer (0.05 M phosphate buffer containing 0.1M L-lysine [pH 7.4], 2 mg/mL NaIO4, and 10 mg/mL paraformaldehyde) overnight at 4 °C. Tissue was equilibrated sequentially in 10%, 20%, and 30% sucrose solutions for 2 h each, before embedding in OCT (ThermoFisher Scientific) and rapidly frozen on dry ice and stored at −80 °C. 10 μm tissue sections prepared using a cryostat were incubated in blocking buffer (PBS + 2% FBS + 1% BSA) for 2 h and then incubated with anti-GFP-AlexaFluor 488 (1:200, clone FM264G), anti-CD8-AlexaFluor 647 (1:200, clone 53-6.7), anti-CD11c-AlexaFluor 594 (1:200, clone N418), anti-CD45.1-BV421 (1:200, clone A20), from Biolegend, or rabbit anti-cleaved caspase-3 (1:500, Asp175) (5A1E, Cell Signaling) in PBS + 10% blocking buffer overnight. For staining of NDV, anti-NDV rabbit polyclonal serum generated by immunizing rabbit with whole inactivated NDV with Freund’s adjuvant twice in a 2-week interval (Labcorp), was used. Slides were preincubated with PBS-Tween (0.1%) for 10 min prior to the 2 h incubation in blocking buffer, and anti-NDV rabbit polyclonal serum (1:200) was added with the primary monoclonal antibodies. Slides were washed with PBS-Tween (0.1%) and incubated for 1 h with donkey anti-rabbit secondary antibodies (1:500, AlexaFluor 594 or 647, Poly4064, BioLegend). ProLong Gold antifade (ThermoFisher Scientific) was used as a mounting reagent, and images were acquired on a Zeiss LSM780 confocal microscope. Images were analyzed using FIJI.

### RT-qPCR

Total RNAs were isolated from lymphoma cells, infected at described above, using a Qiagen RNeasy Mini Kit (Qiagen) at the indicated time post-infection. For excised tumors, samples were preserved on TRIzol^TM^ (Invitrogen) and RNA purification was performed using Direct-zol^TM^ RNA Miniprep Plus (Zymo Research) following the manufacturer’s instructions. cDNA synthesis was performed using the Maxima First Strand cDNA Synthesis Kit for RT-qPCR (Thermo Scientific). Mean fold expression levels of cDNA from three individual biological samples, were normalized to 18 S rRNA levels and calibrated to mock-treated samples according to the 2 − ΔΔCT method.

Data was visualized using Morpheus, https://software.broadinstitute.org/morpheus. Human and murine primer sequences have been compiled in Supplementary Table 3.

### In vitro JEDI killing assays

Single cell suspensions were prepared from spleen and lymph nodes of JEDI mice, and CD8^+^ T cells were negatively selected using MagniSort Mouse CD8^+^ T Cell Enrichment Kit (ThermoFisher Scientific) according to the manufacturer’s protocol. To analyze proliferation, some experiments were performed with splenocytes stained with CellTrace Violet (ThermoFisher Scientific) according to the manufacturer’s protocol. T cell killing assays were performed by co-culturing 10,000 GFP^+^ A20 cells, 10,000 mCherry^+^ A20 cells and naïve bulk JEDI splenocytes (splenocyte:tumor ratios of 5:1 to 20:1) or isolated JEDI CD8^+^ T cells (T cell:tumor ratios of 1:1 to 5:1) in 96-well U-bottom plates. Cells were harvested for analysis by flow cytometry after 2–5 days of culture.

### Human in vitro killing assay with CD3/CD19 bispecific T cell engager

PBMCs were isolated from healthy donor blood by density gradient centrifugation using Ficoll Paque Plus (GE Healthcare). CD8^+^ T cells were negatively selected using MojoSort Human CD8 T cell Isolation Kit (BioLegend) according to the manufacturer’s protocol. PBMCs or CD8^+^ T cells were co-cultured with uninfected or NDV-preinfected SUDHL4 cells (at a 10:1 ratio for PBMCs and 2:1 for CD8^+^ T cells) in the absence or presence of 1.2 ng/ml Blinatumomab (Amgen) for 72 h, followed by flow cytometry analysis. PBMCs/CD8^+^ T cells were labeled with CellTrace Violet prior to seeding for proliferation analyses. For tumor killing quantification, a homogeneous suspension of Precision Counting Beads (BioLegend) was added to each sample prior to analysis by flow cytometry. Normalized cell counts were calculated by dividing the number of event counts of a population of interest by the number of event counts of beads within the same sample.

### Mouse T cell and DC activation and DC tumor Ag uptake assays

Splenocytes from untreated or Flt3L-treated mice were co-cultured with uninfected or NDV-preinfected GFP^+^ A20 cells at a 4:1 or 1:1 ratio for DC activation and tumor Ag uptake analysis, respectively, for 24 h. DC activation was analyzed by a 25-color spectral flow cytometry panel by staining with the following antibodies: CD132-PE, CD80-PE-Cy7, CD16.2-Pacific Blue, Sca-1-AlexaFluor 700, CCR7-PE-Cy5, MHC1b Qa-2-AlexaFluor 647, TCRβ-BV421, CD49b-BV421, CD317-BV605, F4/80-BV510, XCR1-BV650, PD-L1-BV711, CD25-BV785, Ly6G-BV570, Ly6C-PerCP-Cy5.5, CD169-PEDazzle-594, B220-BV750, CD11b-APC-Cy7, I-Ad-FITC, CD8-Pacific Orange, CD86-BV480, Galectin-9-PerCP-eFluor710, and CD11c-AlexaFluor 532 (details are listed in Supplementary Table 2). Viability was assessed by 7AAD. For T cell activation and T cell and DC blockade experiments splenocytes from Flt3L-treated mice were co-cultured with uninfected or NDV-preinfected A20 cells at a 4:1 ratio. Where indicated, splenocytes were treated with 20 μg/ml Clec9A-blocking antibodies (7H11, BioXCell), 20 μg/ml IFNAR-blocking antibodies (MAR1-5A3, BioXCell) or isotype control antibodies 1 h before co-culture, or 1 μM of the AXL inhibitor R428 (S2841, Selleckchem) 24 h and 30 min before co-culture with A20 cells. Blockade experiments and tumor Ag uptake experiments were analyzed by conventional flow cytometry.

### Human monocyte-derived DC activation assay

CD14^+^ monocytes sorted with a CD14 Selection Kit (Mojosort, Biolegend) from PBMCs isolated from healthy volunteers were cultured in 24-well plates at a density of 250,000 cells/ml with 5 ng/ml human recombinant GM-CSF and 20 ng/ml human recombinant IL-4 (Peprotech) for 6 days; medium was replaced after 3 days. After 6 days, the monocyte-derived DCs (moDCs) were co-cultured with uninfected or NDV-preinfected (at 1 and 10 MOI) GFP^+^ SUDHL4 cells for 24 h. DCs were then analyzed by conventional flow cytometry for expression of DC markers and MHC and co-stimulatory molecules.

### Patient DC activation and tumor Ag uptake assay

Cryopreserved PBMCs isolated, pre- and post- Flt3L-treatment, from patients with advanced-stage iNHL that received Flt3L treatment as part of an ISV clinical trial (NCT01976585), were thawed and co-cultured with uninfected or NDV-preinfected GFP^+^ SUDHL4 cells. After 24 h of co-culture, DC activation and tumor Ag (GFP) uptake was analyzed by a 20-color spectral flow cytometry panel by staining with the following antibodies: CD80-BV421, CD19-Pacific Blue, HLA-ABC-BV510, CD14-BV570, CD141-BV605, CD123-BV650, CD40-BV711, CD25-BV785, CD11c-FITC, CD56-PerCP-Cy5.5, CD1c-PerCP-eFluor710, PDL1-PE, CD86-PE-Dazzle594, CD3-PE-Cy5, HLA-DR-PE-Cy7, CD11b-APC, CD83-AlexaFluor 647, CD16-AlexaFluor 700 (details are listed in Supplementary Table 2). Viability was assessed by FVS780 staining.

### Mouse ex vivo cross-presentation assay

B2m^–/–^ GFP^+^ A20 or B2m^–/–^ A20 (Ag-negative) cells were infected with NDV at 1 or 10 MOI. After 24 h, splenocytes obtained from Flt3L-treated or naïve Wt mice were added to the NDV-preinfected lymphoma cells at a ratio of 1:1. After 48 h, splenocytes were isolated form JEDI mice, stained with CellTrace Violet according to the manufacturer’s instructions and added to the culture at a ratio of 5:1. Activation of GFP-specific CD8^+^ T cells was determined after 4 days by analyzing proliferation and cytokine production using flow cytometry. CD11c^+^ APC-depleted and B220^+^Ly6C^hi^ pDC-depleted (pDCs were confirmed to be CD317^+^ and CD11c^low^I-Ad^low^) populations were isolated from Flt3L-treated splenocytes by FACS sorting. For MHC I- and IFN I-blocking experiments, 200 ug/ml anti-H2 (Clone M1/42.3.9.8, BioXCell) or 20 μg/ml IFNAR- (MAR1-5A3, BioXCell) blocking antibodies, or corresponding isotype control (BioXCell) were added before co-culture.

### Human in vitro superantigen assay

PBMCs from healthy donors, isolated as described above, were co-cultured with uninfected or NDV-preinfected SUDHL4 cells (at a 2:1 ratio) in the absence or presence of 10 ng/mL staphylococcal enterotoxin B (Toxin Technology) for 72 h before harvesting for analysis of T cell activation by flow cytometry.

### NDV-reactivity assay

NDV was UV-inactivated by exposure to 120 mJ/cm^2^ for 2 min by using a Stratalinker 2400 UV cross-linker. Inactivation was confirmed by infecting A20 cells with active or UV-inactivated NDV (iNDV), at an MOI of 1 and 10, and analyzing viability and NDV-infection by flow cytometry after 2 h. CD11c^+^ DCs from splenocytes from Flt3L-treated Balb/c mice were isolated with the Magnisort Mouse CD11c positive selection kit (Invitrogen). DCs were resuspended to 5 × 10^6^/ml, plated in 96-well round-bottom plates and pulsed with iNDV at 1 or 10 MOI. After overnight culture, DCs were resuspended to 1 × 10^6^/ml in complete media for co-culture with TdLN cells from Wt or Batf3^−/−^ mice treated as indicated elsewhere. iNDV-pulsed DCs and TdLN cells were co-cultured at a ratio of 1:5 and T cells were analyzed after 24 h by flow cytometry.

### Adoptive transfer of JEDI CD8^+^ T cells

Single cell suspensions were prepared from spleen and lymph nodes of JEDI transgenic mice and stained with CellTrace Violet, and CD8^+^ T cells were isolated by magnetic separations. 1 × 10^6^ CD8^+^ T cells were transferred into Wt or Batf3^−/−^ Balb/c mice by tail vein injection. Tumor and TdLN cells were analyzed at the time points indicated in the figures. Transferred cells were detected by CD45.1 and H2K^d^-GFP_200–208_ tetramer staining.

### Exome and RNA sequencing

Next-generation sequencing and data processing of A20 cancer cell line were performed as previously described^70^. In brief, total DNA and RNA were purified from triplicates of cultured A20 lymphoma cell line using DNeasy Blood and Tissue Kit (QIAGEN) and RNAeasy Mini Kit (QIAGEN). Exome capture was performed using the SureSelectXT mouse exon kit (Agilent). Exome capture libraries were then paired-ended sequenced on a HiSeq 4000 (Illumina) using the HiSeq 4000 sequencing Kit (200 cycles). 50 M exome reads were sequenced from each sample. 500 ng of total RNA per sample was used to generate barcoded mRNA-seq cDNA libraries using TruSeq V2 kit (Illumina). All libraries were sequenced on an Illumina HiSeq4000. 30 M reads were sequenced from each sample.

### Alignment

Both DNA and RNA sequencing data were analyzed using HTSeqGenie^71^ in BioConductor. First, reads with low nucleotide qualities (70% of bases with quality <23) or matches to adapter sequences were removed. RNA reads matching to rRNA (rRNA contigs from GENCODE M15 were also removed. The remaining reads were aligned to the mouse reference genome (GRCm38.p5) using GSNAP^72^ version ‘2013-10-10-v2’, allowing a maximum of two mismatches per 75 base sequence. DNA alignment parameters were: ‘-M 2 -n 10 -B 2 -i 1 –pairmax-dna=1000 –terminal-threshold=1000 –gmap-mode=none –clip-overlap’ and RNA alignment parameters were: ‘-M 2 -n 10 -B 2 -i 1 -N 1 -w 200000 -E 1 –pairmax-rna=200000 –clip-overlap.’

### Variant calling

Somatic variants were called using the union of Lofreq 2.1.2^73^ and Strelka1.0.14^74^ SNV calls, and only the Strelka1 indel calls. For Lofreq2, indel qualities were assigned to the alignments using ‘lofreq indelqual –dindel,’ and somatic mutations were called using ‘lofreq somatic’ with the ‘–call-indels’ option. Strelka-based somatic mutations were called using the Strelka-provided configuration file strelka_config_bwa_default.ini, with the only modification being the setting ‘isSkipDepthFilters = 1’ instead of ‘isSkipDepthFilters = 0.’

### Variant annotation

Somatic mutations were annotated for effects on transcripts using the Ensembl 90 Variant Effect Predictor (VEP)^75^ on GENCODE M15-basic based gene models. The ‘downstream’ plugin^76^ was used in VEP to identify potentially expressed sequence that was downstream of frameshift indels, as well as downstream of stop loss mutations. To identify nonsynonymous mutations, mutations were only retained if their consequence was among the following: frameshift_variant, stop_lost, stop_gained, start_lost, initiator_codon_variant, inframe_insertion, inframe_deletion, missense_variant, coding_sequence_variant, or protein_altering_variant.

### Neoepitope prediction

Expressed mutations were identified by tallying RNA-seq alignments for identified mutations in the exome data, using the tallyVariants function from the R package VariantTools 1.20.0^77^, combined with gmapR 1.20.1^78^. Only RNA reads having a mapping quality > = 23 were tallied. The neoepitope potential of each mutation was predicted after specifying the MHC-I genotype of the A20 cells (assumed to be the same MHC haplotype as BALB/c from which it was derived) and assigning the optimal MHC-neoepitope pair across all MHC-I alleles and 8- to 11-mer peptides containing the mutation. The score per MHC-neoepitope pair was based on the MHC-allele-specific percentile rank of the neoepitope’s IC50 score; this rank was predicted by the NetMHCpan4.0^79^ ‘rank’ method (via IEDB 2.19)^80^. The A20 MHC-I genotype was assumed to be ‘H-2-Kd,’ ‘H-2-Dd,’ and ‘H-2-Ld.’

Peptides (25-mers) used for initial screening were synthesized and purified using PepPower Peptide Synthesis Platform (GenScript). Peptide quality was assessed by mass spectrometry and HPLC to guaranty >75% purity and solubility of individual peptide was tested to determine adequate solvents. 8-11-mer Lrrk1_mut_ peptides were ordered form Genscript (Details are found in Supplementary Table 1 and Supplementary Fig. 7d).

### T cell neoepitope-reactivity assay

For screening, peptides were grouped into peptide pools, each containing 10-11 peptides, and pulsed onto CD11c^+^ DCs isolated from splenocytes from Flt3L-treated mice as described above. All peptides, in pools as well as individually tested, were used at a final concentration of 20 μg/ml. Non-pulsed, DMSO-treated and GFP-peptide (1 μg/ml)-pulsed DCs were cultured in parallel for use as control; since the DMSO control did not differ from the non-pulsed DCs, the non-pulsed DCs are shown as control in all figures. After overnight incubation, DCs were co-cultured with cell suspensions prepared from TdLNs from Flt3L- and NDV-treated mice and analyzed for IFN-γ production after 24 h, as described for the iNDV-reactivity assay.

### Statistical analyses

Data analysis was performed using GraphPad Prism 9. Unpaired two-tailed Student’s t-test was used to compare two independent groups with paired data. Kruskal Wallis and Dunn’s multiple comparison test, or one-way ANOVA or two-way ANOVA were used to compare multiple (>2) groups with one or two independent variables, respectively; with multiple comparisons tests as indicated. *p* values > 0.05 were considered statistically non-significant (ns).

### Reporting summary

Further information on research design is available in the Nature Portfolio Reporting Summary linked to this article.

## Supplementary information

Supplementary Information

Reporting Summary

## Data Availability

Whole exome and RNA sequencing data have been deposited in the Sequence Read Archive (SRA) under accession code PRJNA896242 (https://www.ncbi.nlm.nih.gov/bioproject/PRJNA896242). The remaining data in this study are available in the manuscript, the supplementary materials or available from the corresponding author upon reasonable request. All requests for data and materials will be promptly reviewed by the Icahn School of Medicine at Mount Sinai to verify whether the request is subject to any intellectual property or confidentiality obligations. Patient-related data were generated as part of a clinical trial and may be subject to patient confidentiality. Any data that can be shared will be released via a Material Transfer Agreement. Source data are provided with this paper.

## Source data

Source Data
