# Supplementary material for: Expanding cross-presenting dendritic cells enhances oncolytic virotherapy and is critical for long-term anti-tumor immunity
Source: Nat Commun. 2022 Nov 22;13:7149. doi: 10.1038/s41467-022-34791-8 (PMC9684150; doi:10.1038/s41467-022-34791-8)
Supplement: Supplementary file 1 — Supplementary Information [file 41467_2022_34791_MOESM1_ESM.pdf]

Expanding cross-presenting dendritic cells enhances oncolytic virotherapy and is critical for long-term anti-tumor immunity

Supplementary Figure 1. NDV enhances immunogenicity and susceptibility of tumor cells to T cell-mediated killing.

Supplementary Figure 2. DC activation and Ag-uptake upon Flt3L and NDV treatment.

Supplementary Figure 3. Cross-priming is dependent on type I IFN signaling.

Supplementary Figure 4. Characterization of the myeloid compartment upon Flt3L+NDV treatment.

Supplementary Figure 5. Characterization of immune cells and safety assessment upon Flt3L+NDV treatment.

Supplementary Figure 6. Characterization of T cells upon Flt3L+NDV treatment.

Supplementary Figure 7. Tumor Ag-specific T cell responses upon combination therapy.

Supplementary Figure 8. Batf3-DCs are critical for the anti-tumor effects of Flt3L+NDV.

Supplementary Figure 9. Example gating strategies for flow cytometry data.

Supplementary Table 1. Neoepitope peptides.

Supplementary Table 2. Antibodies used for conventional and spectral flow cytometry.

Supplementary Table 3. RT- qPCR primer sequences (5’ to 3’ end).

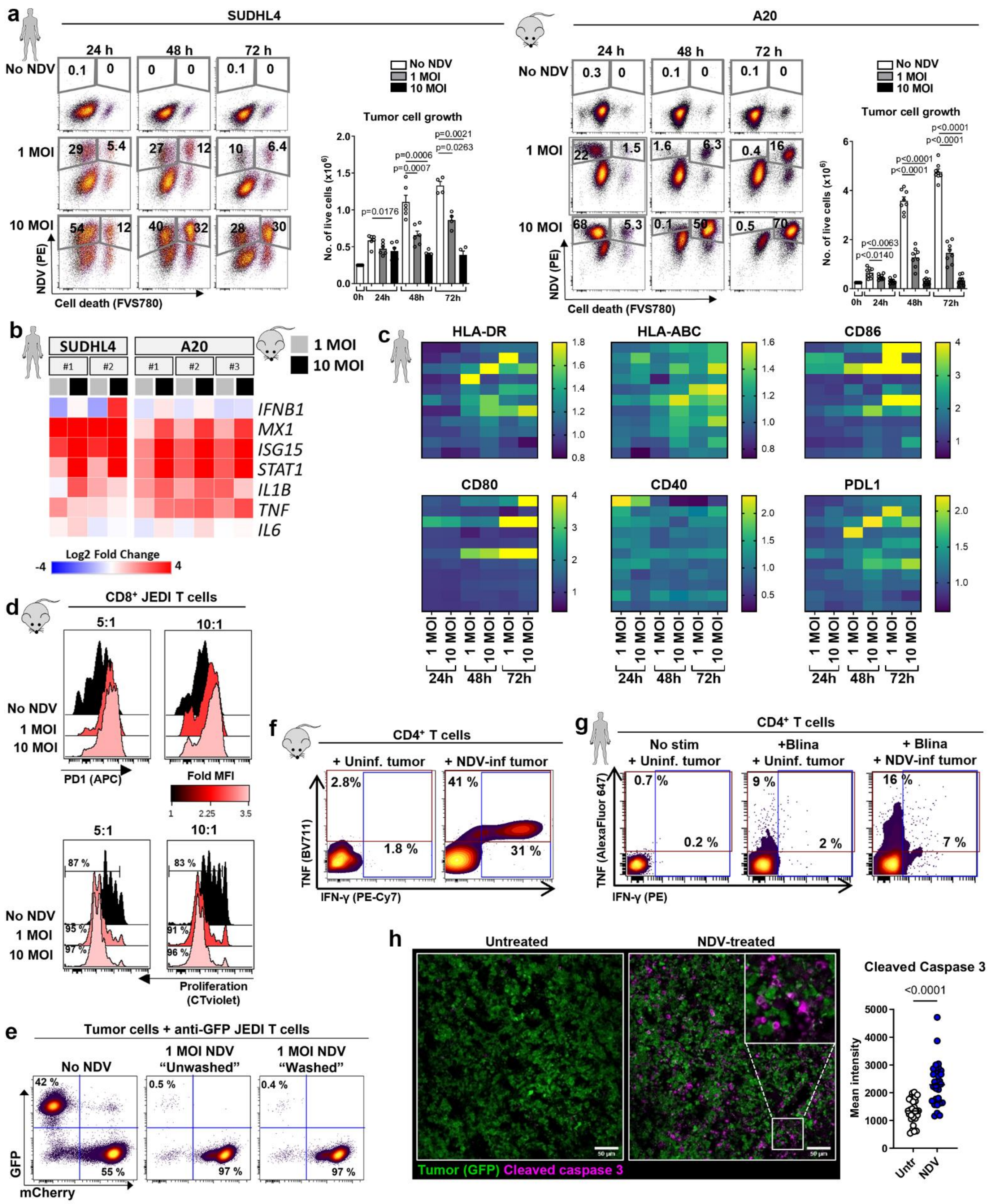

Supplementary Figure 1. NDV enhances immunogenicity and susceptibility of tumor cells to T cell-mediated killing. a. Representative dot plots showing NDV-infectivity and cell death in human SUDHL4 and murine A20 cells, and the effect on tumor cell growth (bar graphs), shown as number of surviving cells in culture, 24h, 48h and 72h post NDV infection. Repeated measures One-way ANOVA with Dunnett's multiple comparisons test, n=3 (SUDHL4) and n=4 (A20) independent experiments, each performed in duplicates. **b.** Heat

map of interferon stimulated and proinflammatory gene expression in human (SUDHL4, n=2) and mouse (A20, n=3) lymphoma cell lines 8h post NDV infection. **c.** Heat map showing expression (analyzed by flow cytometry) of MHC and co-stimulatory molecules on selected patient lymphoma cells (total, n=11; MCL, SLL/CLL and FL, n=3; DLBCL, n=2) 24h, 48h and 72h post NDV infection. Data shows the fold MFI compared to the No NDV condition. **d-f**. Uninfected or NDV-preinfected GFP$^+$ and mCherry$^+$ A20 cells (ratio 1:1) were cocultured with naïve JEDI splenocytes (at 5:1 or 10:1 splenocyte:tumor ratio). **d.** Representative histograms showing PD1 expression (n=5) and proliferation (n=2) in CD8$^+$ JEDI T cells analyzed after 5 days of co-culture. **e**. Contour plots showing tumor killing (day 5) in co-cultures where free NDV was removed ("washed") or not ("unwashed") prior to splenocyte co-culture. Representative of 3 independent experiments. **f.** Contour plots showing activation of CD4$^+$ T cells upon 5 days of co-culture with NDV-infected tumor cells. Representative of 3 independent experiments. **g.** Uninfected or NDV-preinfected human SUDHL4 cells were co-cultured with PBMCs in the absence or presence of the CD3-CD19 bispecific T cell-engager Blinatumomab (Blina). Representative contour plots (of n=4) show activation of CD4$^+$ T cells. **h.** GFP$^+$ A20 tumor-bearing mice were treated with intratumoral NDV and tumors were harvested and cryopreserved after 24h. Representative confocal images and quantification of cleaved caspase 3 (mean pixel intensity), from a total of 12-18 20x images per mouse (n=2); unpaired two-tailed t-test. All data are presented as mean ± standard deviation. MCL, mantle cell lymphoma; SLL, small lymphocytic lymphoma; CLL, chronic lymphocytic leukemia; FL, follicular lymphoma; DLBCL, diffuse large B cell lymphoma.

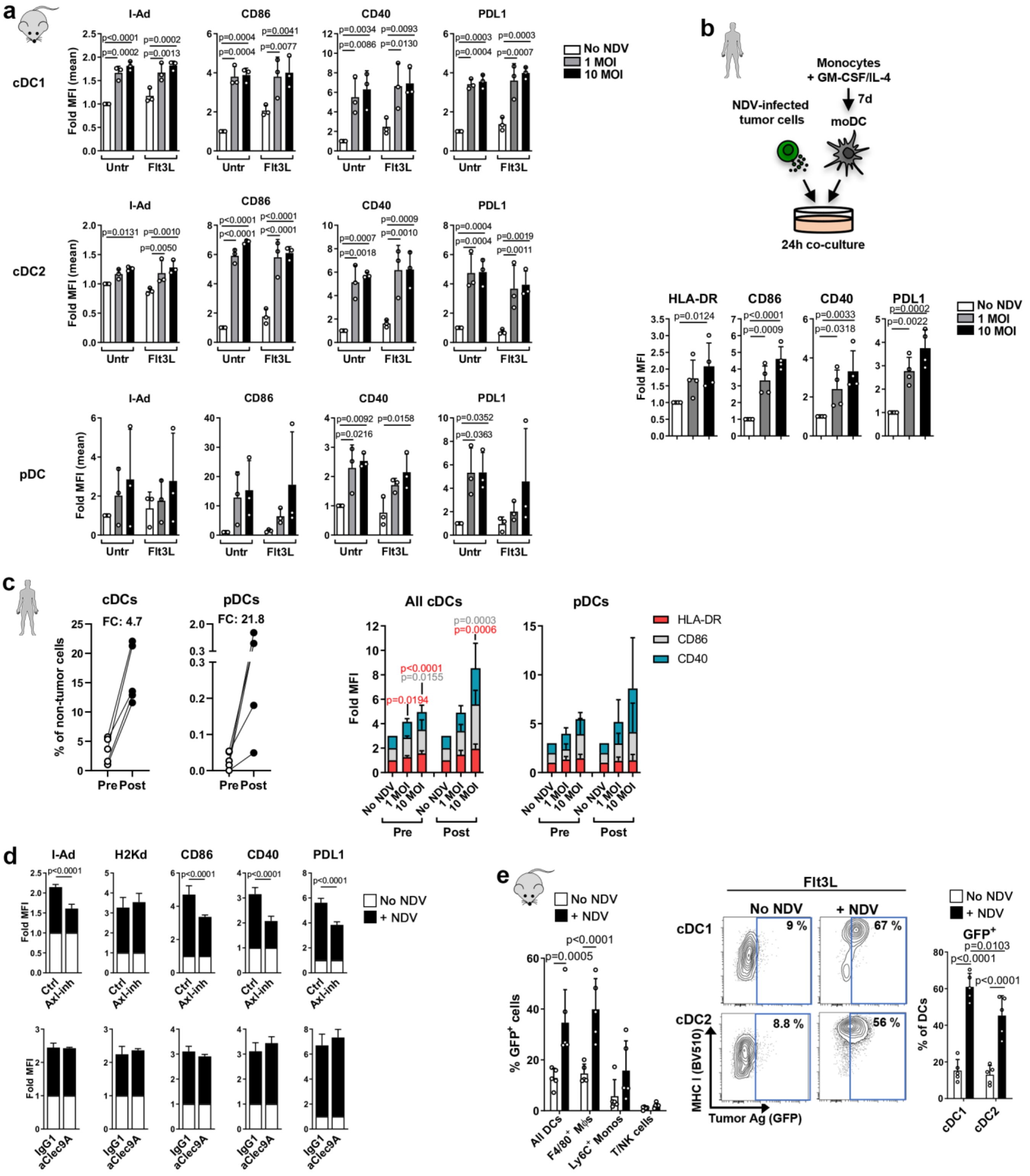

Supplementary Figure 2. DC activation and Ag-uptake upon Flt3L and NDV treatment. **a.** Uninfected or NDV-preinfected A20 cells were co-cultured with splenocytes from untreated or Flt3L-treated Balb/c mice and analyzed after 24h with spectral flow cytometry. The bar graphs show expression of activation markers on cDC1 (XCR1$^+$), cDC2 (CD11b$^+$) and pDC (B220$^+$Ly6C^{hi}) subpopulations. Repeated measures Two-way ANOVA with Dunnett's multiple comparisons test, n=3. **b.** CD14$^+$ monocytes isolated from PBMCs from healthy volunteers were treated with IL-4 and GM-CSF for 7 days to generate monocyte-derived DCs (moDCs). The moDCs were co-cultured with NDV-preinfected human SUDHL4 lymphoma cells and analyzed after 24h for MHC and co-stimulatory marker expression by flow cytometry.

Repeated measures One-way ANOVA with Dunnett's multiple comparisons test (n=4). **c.** The left graphs show the proportion of lineage (CD14, CD3, CD19, CD16, CD56)-negative HLA-DR^+CD11c^+ cDCs and $CD11c^-CD123^+$ pDCs in samples from lymphoma patients before (Pre) and after (Post) Flt3L treatment. Stacked bar graphs (right) showing fold expression (compared to the No NDV condition) of activation markers on cDCs and pDCs after co-culture with NDV-preinfected SUDHL4 cells. One-way ANOVA with Dunnett's multiple comparisons test (n=5). **d.** Cells were cultured as in (**a**) with or without 1 μM of the Axl inhibitor R428, or 20 μg/ml Clec9A-blocking or isotype control antibodies, and cDCs ($Lin^-CD11c^+I\text{-}Ad^+$) were analyzed by flow cytometry for activation markers. The graphs show No NDV vs 10 MOI data analyzed in triplicates, representative from 2 independent experiments; One-way ANOVA with Dunnett's multiple comparisons test. **e.** Splenocytes from untreated and Flt3L-treated mice were co-cultured with uninfected or NDV-preinfected GFP^+ A20 cells (1:1 ratio) for 24h and analyzed by flow cytometry. The bar graphs show tumor antigen (GFP)-uptake in different immune subpopulations (left) and in cDC1 ($XCR1^+$) and cDC2 ($CD11b^+$) subpopulations (contour plots and bar graphs to the right). Two-way ANOVA with Bonferroni's multiple comparisons test, n=5. All data are presented as mean ± standard deviation.

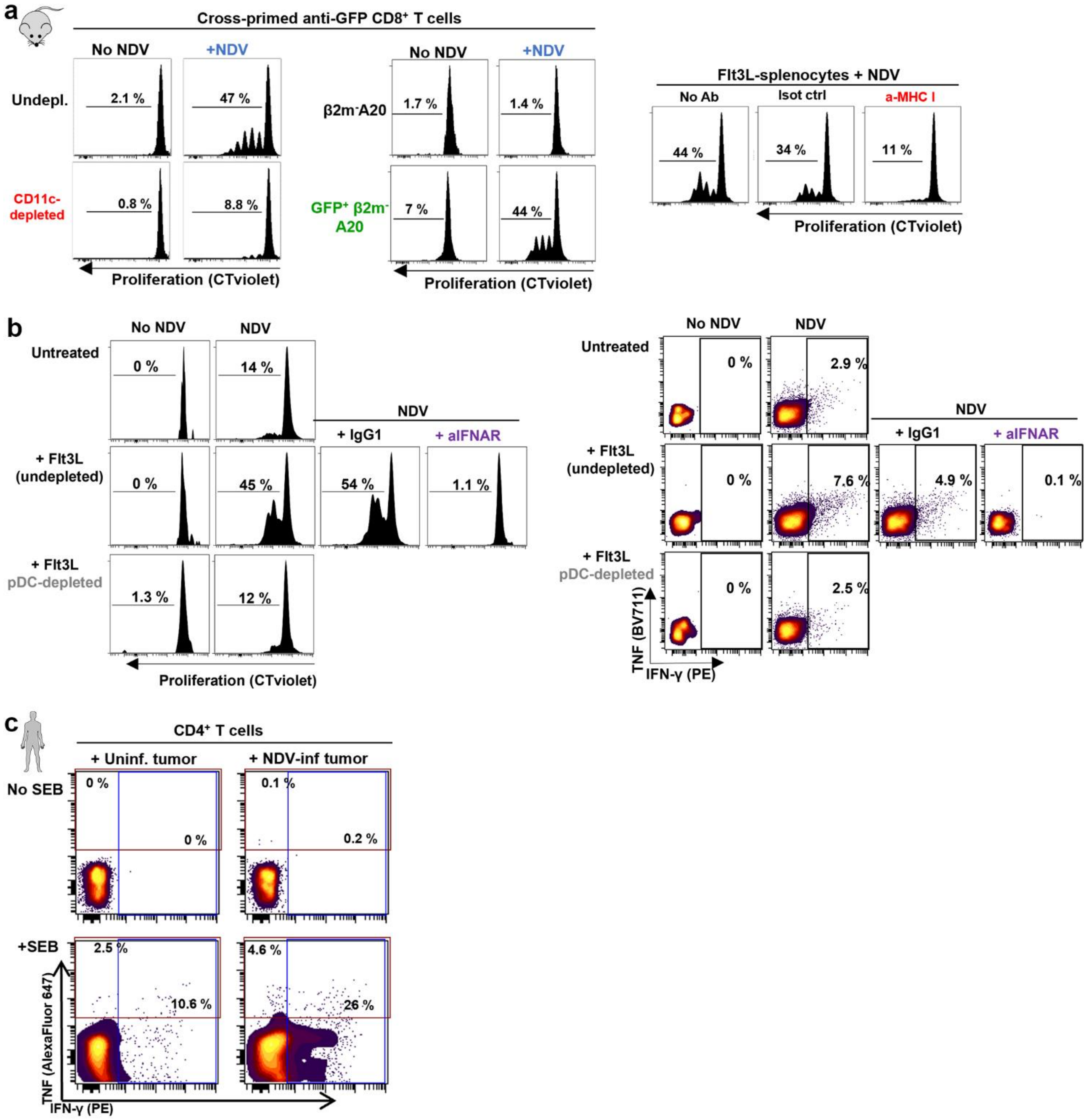

Supplementary Figure 3. Cross-priming is dependent on type I IFN signaling.

a-b. NDV-preinfected MHC I-deficient β2m$^{-/-}$ A20s or β2m$^{-/-}$ GFP^{+} A20s were co-cultured with bulk splenocytes from untreated or Flt3L-treated mice for 48h and CellTrace violet-stained anti-GFP JEDI T cells were added to the co-cultures. For testing APC-dependency, CD11c-depleted splenocytes were used, and for MHC I-dependence, MHC I (H2)-blocking or isotype control antibodies were added before co-culture (a). For pDC-dependency, splenocytes were depleted of B220^{+}Ly6C^{hi} pDCs, and for type I IFN-dependence, IFNAR-blocking or isotype control antibodies were added before co-culture (b). The representative histograms show CD45.1^{+} CD8^{+} T cell proliferation (a and b, left panel) or cytokine production (b, right panel) after 3-4 days. Representative from n=2 (CD11c-depletion), n=3 (anti-MHC-I), n=7 (β2m$^{-/-}$ A20s vs β2m$^{-/-}$ GFP^{+} A20s) or n=2 (pDC-depletion and anti-IFNAR) performed in duplicates. **c.** NDV-preinfected SUDHL4 cells were co-cultured with PBMCs in the absence or presence of Staphylococcal enterotoxin B (SEB) and T cells were analyzed after 3 days. The representative

(of n=5) contour plots show that NDV-infected tumor cells induce TNF and IFN-γ production in $CD4^+$ T cells. All data are presented as mean ± standard deviation.

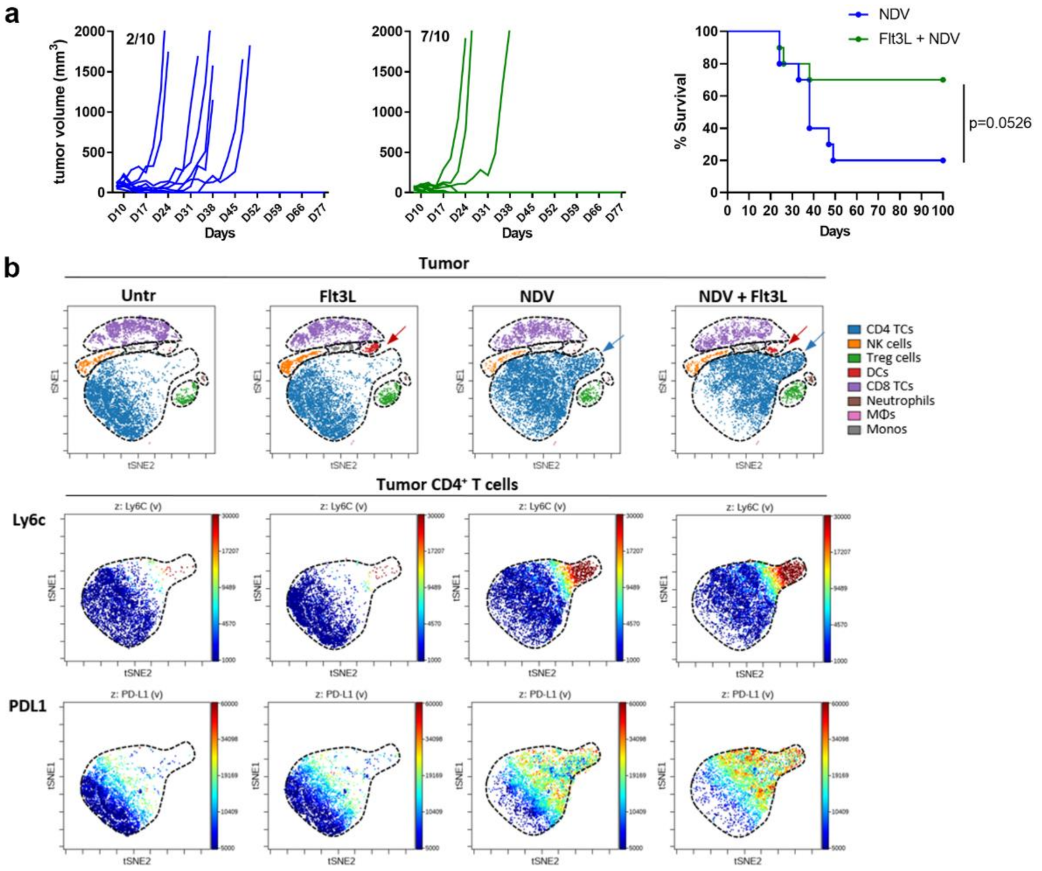

c

	Tumor					TdLNs				
	cDC1	cDC2	DN DCs	pDCs	All DCs	cDC1	cDC2	DN DCs	pDCs	All DCs
Untr vs. Flt3L	0.0073	<0.0001	<0.0001	<0.0001	<0.0001	<0.0001	0.0203	ns	0.0001	<0.0001
Untr vs. NDV	ns	ns	ns	ns	ns	ns	ns	ns	ns	ns
Untr vs. Flt3L+NDV	ns	0.0098	0.0001	ns	ns	<0.0001	ns	ns	ns	0.0002
Flt3L vs. NDV	0.0062	<0.0001	0.0001	<0.0001	0.0001	<0.0001	0.0013	ns	<0.0001	<0.0001
Flt3L vs. Flt3L+NDV	0.0306	0.0029	ns	<0.0001	0.0150	<0.0001	ns	ns	0.0023	<0.0001
NDV vs. Flt3L+NDV	ns	0.0085	0.0016	ns	ns	<0.0001	0.0177	ns	ns	<0.0001

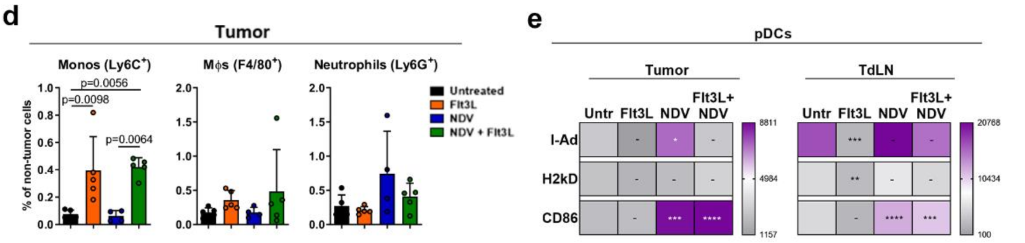

Supplementary Figure 4. Characterization of the myeloid compartment upon Flt3L+NDV treatment. a. GFP+ A20 tumor-bearing mice were treated with 9 doses of Flt3L starting from day 3 and two doses of NDV starting from day 8 post tumor inoculation and followed for tumor growth and survival. Graphs show individual growth curves, and numbers refer to mice with complete remission (CR) vs total number of mice (CR/total) in each group; n=10 mice per group, Log-rank (Mantel-Cox) test (survival). **b-e.** Tumors, TdLNs and spleens from mice treated as in Fig 3c were harvested and analyzed by spectral flow cytometry (n=5) **b.** Representative viSNE plots showing the intratumoral immune cell populations identified (top) and relative expression (color code indicates the mean fluorescence intensity) of Ly6c and PDL1 on the CD4+ T cell population (bottom). **c.** Statistical differences within intratumoral and TdLN DC subsets (shown in Fig 3e) between treatments. One-way ANOVA (All DCs) or Two-way ANOVA (DC subsets) with Tukey's multiple comparisons test. **d.** Proportion of intratumoral monocytes (Monos), macrophages (MΦ) and neutrophils upon treatment. One-

way ANOVA with Tukey's multiple comparisons test. **e**. The heat maps show the relative expression (mean MFI) of activation markers in intratumoral and TdLN pDCs. Two-way ANOVA with Holm-Sidak's multiple comparisons test (vs untreated). All data in bar graphs are presented as mean ± standard deviation. * $p < 0.05$, ** $p < 0.01$, *** $p < 0.001$, **** $p < 0.0001$.

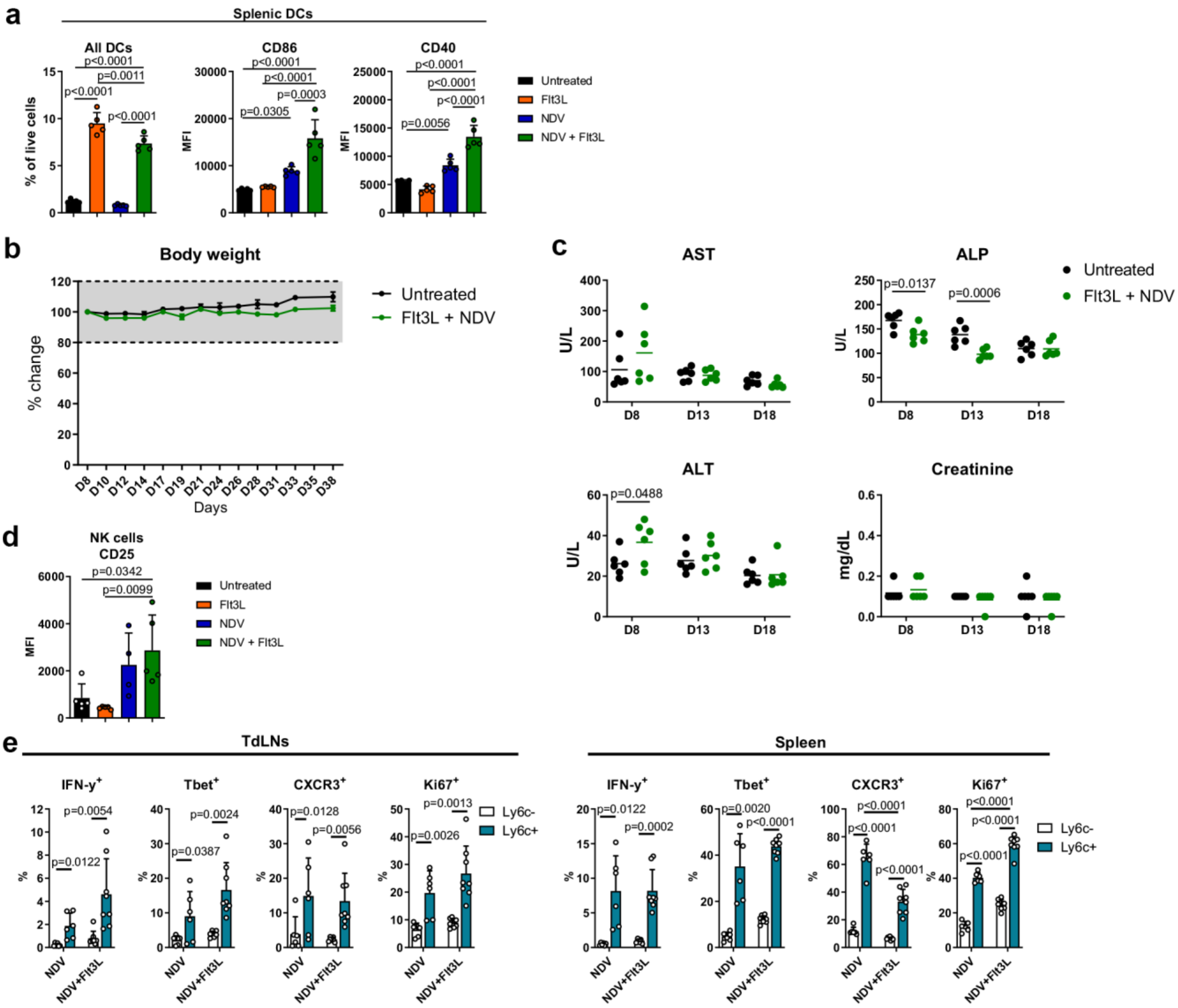

Supplementary Figure 5. Characterization of immune cells and safety assessment upon Flt3L+NDV treatment. a. Proportion and activation of splenic DCs in GFP^{+} A20 tumor-bearing mice treated with Flt3L and NDV as shown in Fig 3c. Analyzed by spectral flow cytometry (n=5). One-way ANOVA with Tukey's multiple comparisons test. **b**. Maintained body weight (percent change) in untreated and Flt3L+NDV-treated tumor-bearing mice. **c.** Analysis of serum liver and kidney enzymes shows slightly elevated ALT levels prior to NDV treatment, which equalizes after initiation of treatment, and a mild improvement (decreased levels) of ALP in the Flt3L+NDV cohort. **b-c**. Time points indicate days post tumor inoculation. Two-way ANOVA with Bonferroni's multiple comparisons test; n=6 mice per group. **d**. Activation of intratumoral NK cells in GFP^{+} A20 tumor-bearing mice treated with Flt3L and NDV as shown in Fig 3c. Analyzed by spectral flow cytometry (n=5). One-way ANOVA with Tukey's multiple comparisons test. **e**. TdLNs and spleens from NDV-alone or Flt3L+NDV-treated GFP^{+} A20 tumor-bearing mice were harvested and IFN-γ, Tbet, CXCR3 and Ki67 were analyzed in Ly6C^{+} vs Ly6C^{-} CD4^{+} T cells. Paired or unpaired (within and between treatment conditions, respectively) two-tailed t-test; NDV, n=6; NDV+Flt3L n=8. Representative from 2 independent experiments. All data in bar graphs are presented as mean ± standard deviation.

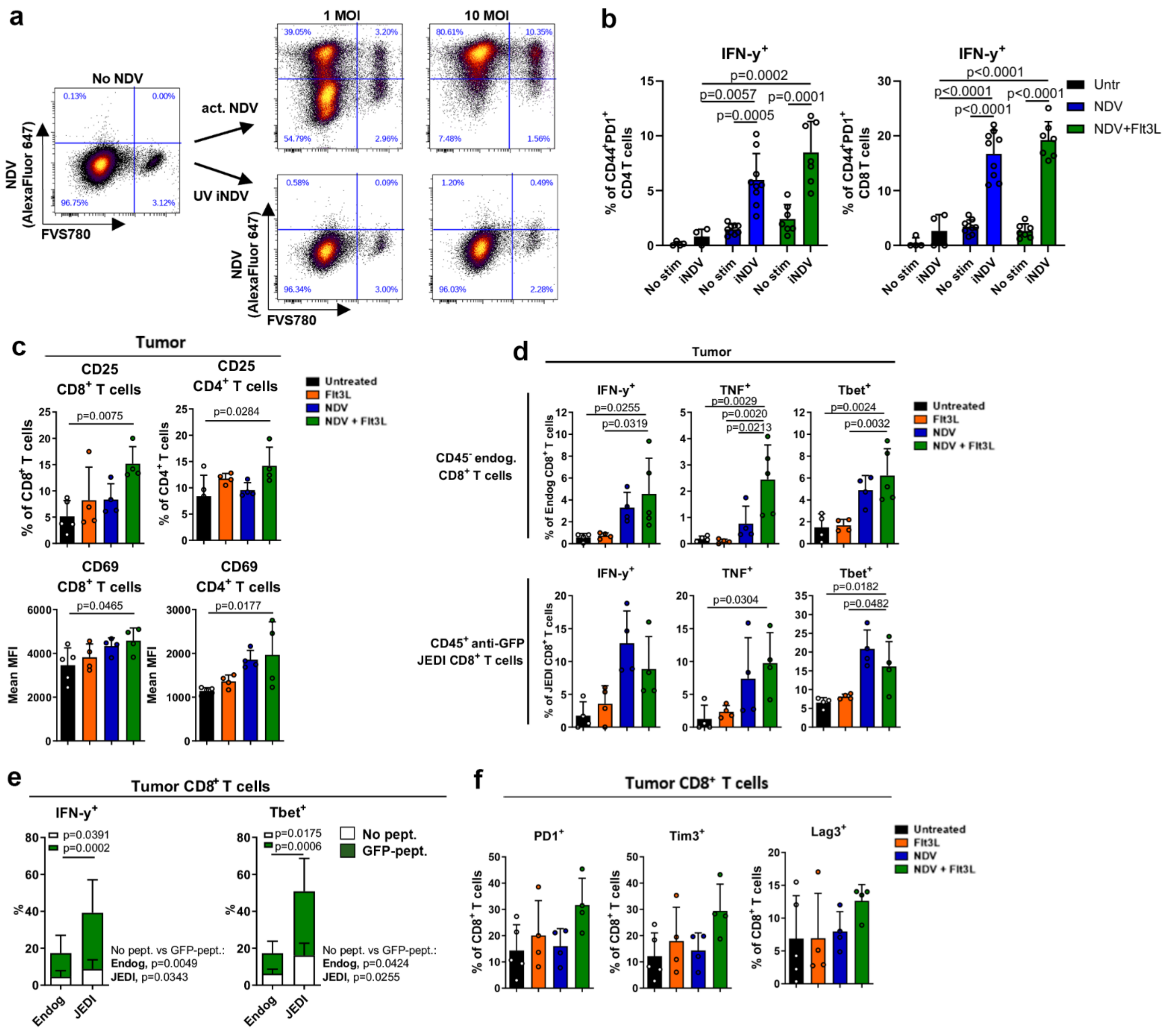

Supplementary Figure 6. Characterization of T cells upon Flt3L+NDV treatment.
a. A20 lymphoma cells were treated with active or UV-inactivated NDV (iNDV), and NDV-infectivity and cell viability was analyzed by flow cytometry after 24h. **b.** TdLNs from untreated (ctrl), NDV- or NDV+Flt3L-treated A20 tumor-bearing mice were harvested and co-cultured with CD11c^{+} DCs: unstimulated (No stim) or pulsed with iNDV. NDV-reactivity in CD44^{+}PD1^{+} CD4^{+} and CD8^{+} T cells (measured as IFN-γ production) was analyzed after 24h. The bar graphs show data from untreated (Untr: n=4), NDV (n=9) and NDV+Flt3L (n=7) groups pooled from 2 independent experiments. Paired, two-tailed t test (No stim vs iNDV) or one-way ANOVA with Tukey's multiple comparisons test for comparison between treatments. **c-f.** GFP^{+} A20-tumor-bearing mice were treated with Flt3L and NDV. 1 × 10^{6} CD45.1^{+} anti-GFP CD8^{+} T cells were adoptively transferred 2 days before NDV treatment and intratumoral T cells were analyzed after 5 days by spectral flow cytometry, n=5 mice per group. Bar graphs showing CD25 and CD69 expression in intratumoral CD8^{+} and CD4^{+} T cells (**c**) and IFN-γ, TNF and Tbet expression in intratumoral endogenous (CD45.1^{-}) and JEDI (tetramer^{+} CD45.1^{+}) CD8^{+} T cells (**d**); one-way ANOVA with Dunnett's multiple comparisons test. **e.** CD19-

depleted tumor samples were treated for 6h with GFP-peptide (or control media, Unstim) and $CD8^+$ T cell IFN-γ and Tbet expression was analyzed by flow cytometry; paired, two-tailed t test. **f.** Bar graphs showing expression of exhaustion markers on intratumoral $CD8^+$ T cells. All data in bar graphs are presented as mean ± standard deviation.

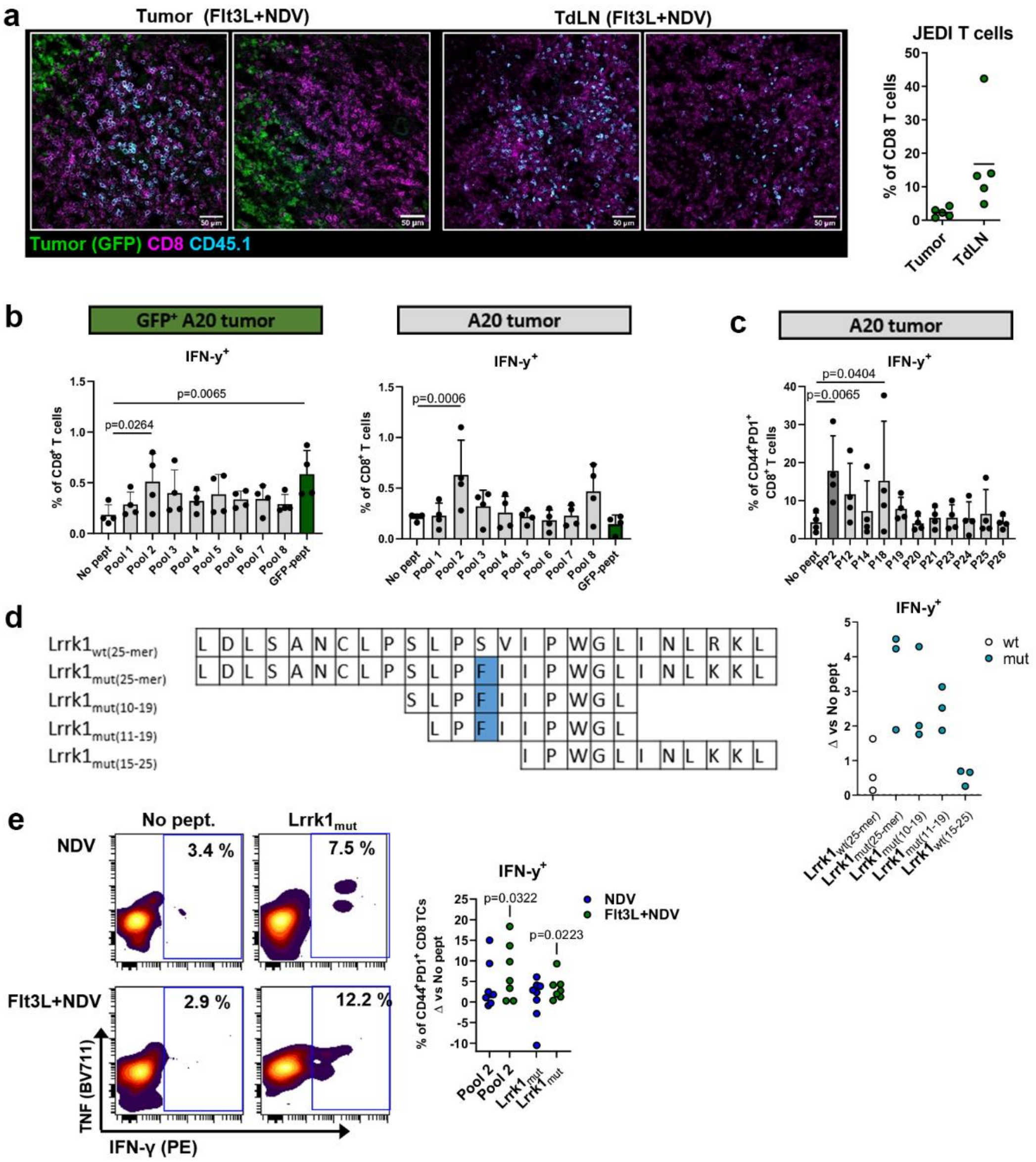

Supplementary Figure 7. Tumor Ag-specific T cell responses upon combination therapy. **a.** GFP$^+$ A20-tumor-bearing mice were treated with Flt3L and NDV as indicated in Fig 3c. 1 × 10^6 CD45.1$^+$ anti-GFP CD8$^+$ T cells were adoptively transferred 2 days before NDV treatment and tumors and TdLNs were analyzed after 5 days by immunofluorescence (left) or by flow cytometry (right). Confocal images of cryopreserved tissues from Flt3L+NDV-treated mice showing infiltration of CD45.1$^+$ JEDI T cells in two different tumor and TdLN areas; images were collected from 3 mice (left). The scatter plots show the proportion of CD45.1$^+$ JEDI T cells within the CD8$^+$ T cell population in the tumor and TdLN as measured by flow cytometry (right); n=5 mice per group. **b-c**. TdLN cells isolated from Flt3L+NDV-treated A20 or GFP$^+$ A20 tumor-bearing mice were cocultured with DCs pulsed with pooled (**b**) or individual (**c**) candidate neoepitope peptides identified by whole-exome and RNA sequencing, or with GFP-peptide. The bar graphs show IFN-γ production in CD8$^+$ T cells (**b**) or in CD44$^+$PD1$^+$ CD8$^+$ T cells (**c**) after 24h co-culture. One-way ANOVA with Dunnett's multiple comparisons test (n=4). **d.** T cell reactivity was further tested against short peptides derived from the immunogenic 25-mer peptide 18 ($Lrrk1_{mut}$) as well as the wild type sequence ($Lrrk1_{wt}$). The cartoon shows the amino acid sequence of the peptides (left) and the bar graphs the IFN-γ production in CD44$^+$PD1$^+$ CD8$^+$ T cells upon co-culture with peptide-pulsed DCs, normalized

to the “No pept” condition (right). **e.** TdLN cells isolated from A20 tumor-bearing mice treated with NDV alone or Flt3L+NDV were co-cultured with peptide pool 2- or $Lrrk1_{mut}$ peptide-pulsed DCs and T cells were analyzed after 24h. Representative contour plots and graph showing IFN-γ production in $CD44^{+}PD1^{+}$ $CD8^{+}$ T cells, normalized to the “No pept” condition. Paired, two-tailed t test (vs No pept.). NDV, n=8; Flt3L+NDV, n=7, pooled data from 2 independent experiments. All data are presented as mean ± standard deviation.

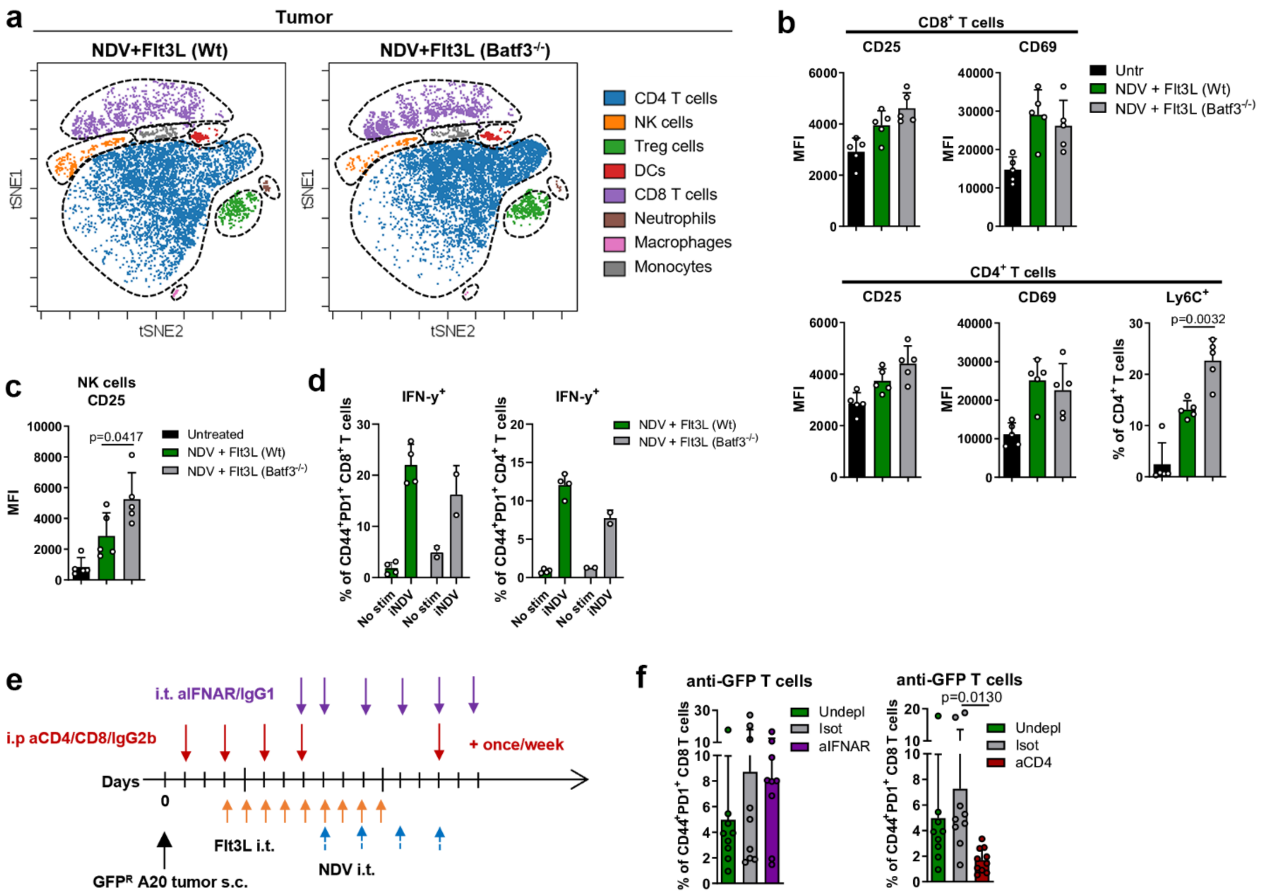

Supplementary Figure 8. Batf3-DCs are critical for the anti-tumor effects of Flt3L+NDV. a-c. GFP$^+$ A20 tumor-bearing Wt and Batf3$^{-/-}$ mice were treated with Flt3L and one dose of NDV as shown in Fig 3c, and tumors were analyzed by spectral flow cytometry after 24h. Representative viSNE plots showing that major intratumoral immune cell populations are not significantly altered (**a**), and bar graphs reflecting maintained Flt3L+NDV-induced early T and NK cell activation (**b-c**) in Batf3$^{-/-}$ mice compared to Wt mice. One-way ANOVA with Tukey's multiple comparisons test; n=5 mice per group. **d.** TdLNs from Flt3L+NDV-treated A20 tumor-bearing Wt (n=4) and Batf3$^{-/-}$ (n=2) mice were harvested and co-cultured with isolated splenic CD11c$^+$ DCs: unstimulated (No stim) or pulsed with UV-inactivated NDV (iNDV). NDV-reactivity in CD44$^+$PD1$^+$ CD4$^+$ and CD8$^+$ T cells (measured as IFN-γ production) was analyzed after 24h. **e.** GFP$^+$ A20 tumor-bearing Wt mice treated with anti-CD8, anti-CD4, anti-IFNAR or isotype control antibodies were treated with Flt3L and NDV as indicated and followed for tumor growth and survival. **f.** Blood was drawn from mice in (**e**) 1 week after completed treatment and analyzed for tetramer$^+$ anti-GFP CD8$^+$ T cells by flow cytometry. Bar graphs shows the percent anti-GFP T cells in the CD44$^+$ PD1$^+$ CD8$^+$ T cell population after intratumoral anti-IFNAR blockade (left) or in CD4-depleted mice (right). n=8 (IgG2b), n=9 (undepleted, IgG1, aIFNAR), n=10 (aCD4). Unpaired, two-tailed t test. All data are presented as mean ± standard deviation.

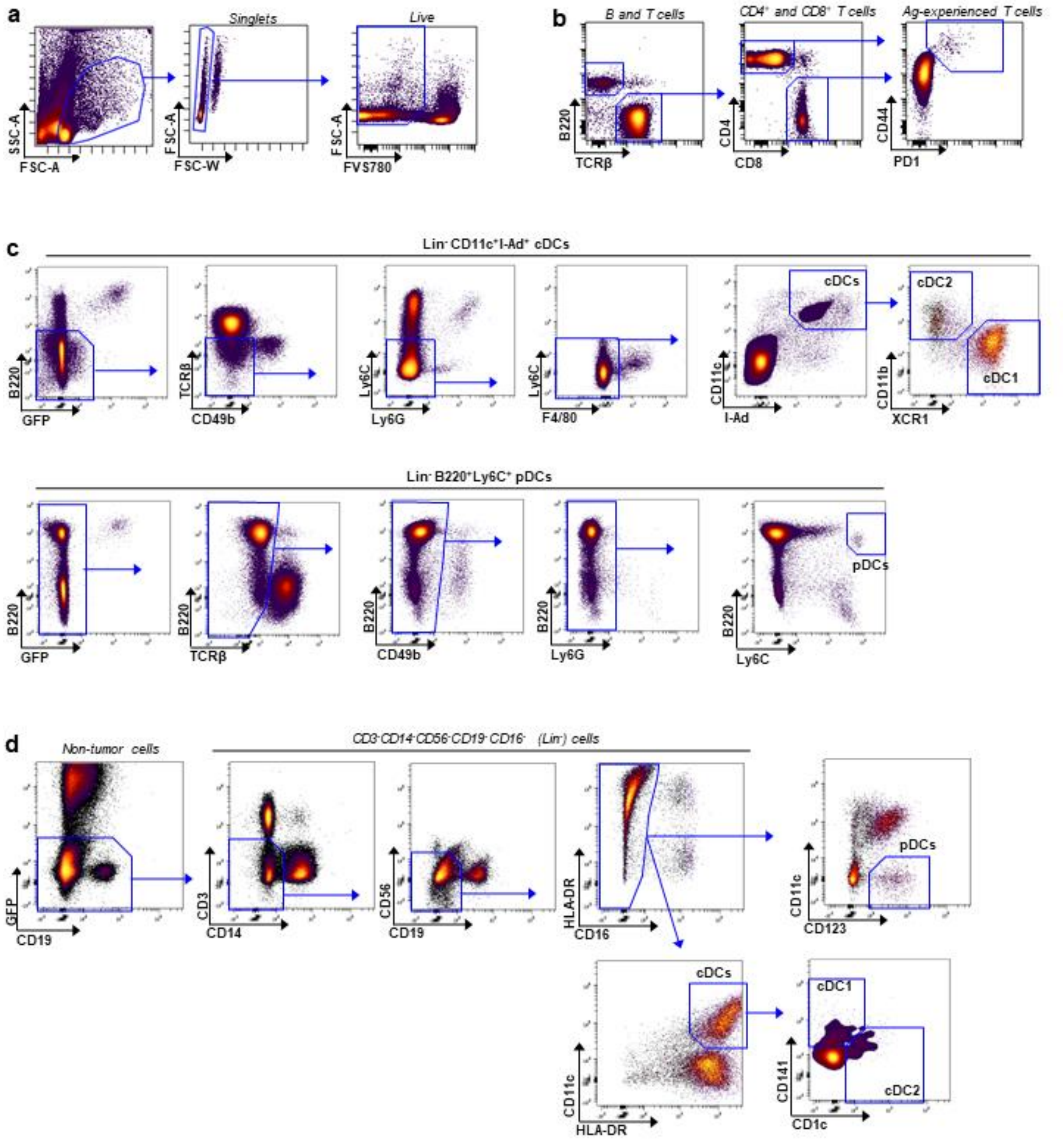

Supplementary Figure 9. Example gating strategies for flow cytometry data. Gating strategy for (**a**) singlets and live cells (used in all analyses), and (**b**) for identifying B220^{+} B cells, TCR-β^{+} CD4^{+} or CD8^{+} T cells, and CD44^{+}PD1^{+} antigen-experienced (CD4^{+} or CD8^{+}) T cells (from live cells). **c.** Gating strategies for murine CD11c^{+}I-Ad^{+} cDCs and subsets (XCR1^{+} cDC1 and CD11b^{+} cDC2 subsets, upper panel) and B220^{+}Ly6C^{+} pDCs (also confirmed to be CD11c^{low}I-Ad^{low} and to express CD317 and CD11b, lower panel). **d.** Gating strategy for human CD11c^{+}HLA-DR^{+} cDCs and subsets (CD141^{+} cDC1 and CD11c^{+} cDC2) and CD123^{+} pDCs. Lin, lineage.

Supplementary Table 1. Neoepitope peptides.

	Peptide number	Neoepitope peptide (mutation)	MHC Allele	Gene
Peptide pool 1	1	YFLNLNSRSPEY**R**SLFIDDKLKKGV	H-2-Dd	Cul3
	3	SKLLIYGRPVYV**P**LIARRSSRFAGT	H-2-Dd	Fig4
	4	QKKSQNLAREER**V**GPPKDLASLGSL	H-2-Dd	Fbf1
	5	TPERREEKGTSP**R**DYRHYLRMWAKE	H-2-Dd	Tbc1d9b
	6	EYTTIHYKYMCN**G**SCMGGMNRRPIL	H-2-Kd	Trp53
	7	ARSTREMPSALS**S**LLLGVAYFKGQW	H-2-Ld	Serpinf1
	8	YASRFIQLVRSK**S**PKITYFTRYAKC	H-2-Dd	Plk4
	9	RQREARKSGSKP**D**NFALISVSPHPS	H-2-Dd	Kmt2e
	10	WLKKQRSIVKNW**P**QRYFVLRAQQLY	H-2-Dd	Arhgap25
	11	LDLMGASQHSLR**P**LSWRRLYLSRAK	H-2-Ld	Orai3
Peptide pool 2	12	CVVWGGASYAVG**V**AALRGPMQLSLA	H-2-Kd	Tmem160
	14	NHEGEVNRARYM**L**QNPHIIATKTPS	H-2-Kd	Rbbp7
	18	LDLSANCLPSLP**F**IIPWGLINLKKL	H-2-Ld	Lrrk1
	19	VVWGGASYAVGL**G**ALRGPMQLSLAG	H-2-Kd	Tmem160
	20	SYVHKVPQFQPF**A**GSSMFAPLKTLP	H-2-Ld	Med13l
	21	LSRAYLDLLTTW**P**TRLHYDLQKGAW	H-2-Ld	Fancf
	23	YSTCSPRKLSPF**C**SFASTELFHFHV	H-2-Ld	Tmem8b
	24	LLGLVHRQDPRF**A**PQAELLLLRGGI	H-2-Ld	Tmem102
	25	DFCRHKVLPQLL**I**AFEFGNAGAVVL	H-2-Ld	Scyl1
	26	EFAEKRRPFQAN**S**ISLSNLVKHLGM	H-2-Ld	Gba2
Peptide pool 3	27	LGDPKPRPLPAC**R**HLSWAKPQPLNE	H-2-Ld	Mbtps1
	28	ETAPRLLRFPWS**L**KLTSSRPPEALM	H-2-Ld	Mark4
	29	ESTRAPLPESIS**L**PQANQAPSLEAM	H-2-Ld	Dnaaf3
	30	FSFIISLLPLLM**F**FHNNMEYMITTW	H-2-Ld	mt-Nd5
	31	IKEWAAYKGKSP**K**TPELVEALAFRE	H-2-Dd	Fam120a
	33	LDASAILDTAKY**G**ALVKVLGKHSRL	H-2-Kd	Lrpprc
	34	GPEVFPMSRLWD**A**RLRHYLGSRYDA	H-2-Ld	Dnaaf3
	35	SLDERLFSPRLA**Q**PVASSQVLIVAA	H-2-Dd	Cbarp
	36	EEDSFSLCFPKR**I**TRNLQKMRMGKT	H-2-Ld	Brca2
	37	PWWRKRFVSAMP**N**APIPFRKKEKQE	H-2-Ld	Gapvd1
Peptide pool 4	38	WIVLREPITVSS**K**QMSHFRTLNFNE	H-2-Kd	Car2
	39	VDRRRQRSIFRA**L**LHFVEGGECEEE	H-2-Dd	Ifrd2
	40	FCYRSYLREHYR**M**HSGEYPYKCEEC	H-2-Kd	Zfp874b
	41	PALTEDGSPTAA**L**GALHSPLPLSPL	H-2-Ld	Wiz
	42	GAPVIYPAASNH**N**SLSFDGGLSGQG	H-2-Kd	R3hdm1
	44	SCRSCSHALAAH**I**SHLENVSEEEMD	H-2-Kd	Kat2b
	45	FGVLLWTKVLGP**F**IGVQVPQEKVER	H-2-Dd	Gstt2
	46	GKSPAQILIRFQ**F**QRNLIVIPKSVT	H-2-Ld	Akr1e1
	47	VPEAPKETPTPQ**W**KGLRSSALRPKR	H-2-Ld	Bptf
	48	SSLSNNSLKSSK**Y**SSLRTTSSTATA	H-2-Kd	Ankrd50
Peptide pool 5	49	WPDGQQDITEVT**N**RPLTAGTLFKNS	H-2-Ld	Myo1g
	50	GYHVSAKCFGYM**M**QQLMNLAGGAVV	H-2-Dd	Hdac7
	51	WAGLTDQHVKLP**L**GMTAENLAAKYN	H-2-Ld	Acaa2
	52	DGLPKNSPNNIS**A**ISNPPGTPRDDG	H-2-Dd	Ssbp3
	53	VKETCAACQKTV**F**PMERLVADKLIF	H-2-Ld	Limd2
	54	LEGLETMRQLRN**P**LRKSTVGRSLKD	H-2-Dd	Mpg
	56	EEVLTVQDLVDF**F**PVYRCLHIYSAL	H-2-Ld	Exoc6
	57	MLLPSDVARL**A**LGYLQQENLTST	H-2-Ld	Npat
	58	EAIYFCALWYSN**L**F	H-2-Kd	Iglv1
	60	ISLPSMIREIFA**A**FTRPLALLYENS	H-2-Ld	Rif1

Peptide pool 6	61	TTISKSGGDYAY**I**LEVYGSLPAFLK	H-2-Dd	Slc7a5
	62	GQGPYQAMPQDM**A**NTPDMFSPDQSS	H-2-Ld	Bcl9l
	64	GGLIILEPRFTG**G**TLAMLLNIPPQK	H-2-Dd	Ppfibp2
	65	GRPDNTGRGYVL**I**RILRRAVRYSHE	H-2-Dd	Aars
	66	IIKEPVPDSGLL**G**LFQGQSPLTSC	H-2-Ld	Isoc2a
	67	RRARDDCVYQVE**R**ERLKLKQLEEDK	H-2-Kd	Cep120
	68	NKEQVSQLLPEK**L**AEQLIRVYCKKK	H-2-Ld	Samhd1
	69	RQNNDSDCGAFV**S**QYCKHLALSQPF	H-2-Ld	Senp3
	70	LCRSKNMPKSTI**K**SALKTEKNKGIY	H-2-Ld	Taco1
	71	ANNPYVLMVLYK**A**KVYNIQIRYQEE	H-2-Kd	Lcp2
Peptide pool 7	72	DATLTQYVKWTN**G**KSLGGIEGCLSK	H-2-Kd	Ttc38
	74	LSQKARQKTDVF**T**PDYIAGVSPFAE	H-2-Kd	Nob1
	76	DPHRRPDFASIL**H**QLEALEAQVLRE	H-2-Ld	Map3k11
	77	GLRSDELLGLTH**A**YSVRWSETSVEH	H-2-Kd	Tm9sf1
	78	LYMGVSSLQGIQ**L**FDRLKLFGMPAK	H-2-Dd	Slc4a8
	79	DKVDLLNQVDWN**A**WLYAPGLPPVKP	H-2-Ld	Lta4h
	80	HILACAPSNSGA**G**LLCQRLRVHLPS	H-2-Ld	Mov10
	81	LHTAGGKGYFDA**P**ALAMDYRSLGFR	H-2-Dd	Hey1
	83	ELDGAVGALMTT**L**GDLGLLEETLVI	H-2-Dd	Arsa
	84	WRDAAYHKSVWR**R**VEAKLHLRRANP	H-2-Kd	Fbxl14
	86	SLGSSPALQLLM**S**TMESETEAAVPE	H-2-Ld	Pprc1
	87	GVYDYLMYVGRV**G**FQVPDWLHHLLM	H-2-Kd	Snx14
Peptide pool 8*	13	SAVPVILPQAPS**A**PSYAIYLQPAQA	H-2-Dd	E2f8
	15	LSISNIQPEDEA**M**YICGVGDTIKEQ	H-2-Kd	Iglv3
	17	MTRQCPPQESG**P**ALSGSVLAEAAV	H-2-Dd	Dolk
	22	SSSGADRYLSIS**D**IQPEDEAIYICG	H-2-Kd	Iglv3
	59	ASFSLGASAKLT**Y**TLSSQHSTYTIE	H-2-Dd	Iglv3
	63	YLSISNIQPEDE**P**IYICGVGDTIKE	H-2-Kd	Iglv3
	75	WLTDNTYKYEDL**L**RVMGEIISALEG	H-2-Kd	Ccnf
	85	GYMDPTEPSFVA**V**VITIVFNPLFWN	H-2-Kd	Pemt
	89	GADRYLSISNIQ**A**EDEAIYICGVGD	H-2-Kd	Iglv3
	90	KHGFFVNPSDSV**G**VIAANIFSIPYF	H-2-Dd	Pgm2

**Dissolved in DMSO*

Supplementary Table 2. Antibodies used for conventional and spectral flow cytometry.

Marker	Fluorophore	Clone	Company	Species	Cat#
CD11c	FITC	B-ly6	BD Biosciences	anti-human	561355
CD123	Brilliant Violet 650	6H6	Biolegend	anti-human	306019
CD14	Brilliant Violet 570	M5E2	Biolegend	anti-human	301832
CD14	FITC	M5E2	Biolegend	anti-human	982502
CD141	Brilliant Violet 605	M80	Biolegend	anti-human	344117
CD16	Alexa Fluor 700	3G8	Biolegend	anti-human	302026
CD19	APC	HIB-19	Biolegend	anti-human	302212
CD19	Pacific Blue	HIB-19	Biolegend	anti-human	302232
CD19	PE-Cy7	HIB-19	Biolegend	anti-human	302216
CD19	PerCP-Cy5.5	HIB-19	Biolegend	anti-human	302230
CD1c	PerCP-eFluor 710	L161	eBioScience	anti-human	46001542
CD20	PE	2H7	BD Biosciences	anti-human	556633
CD25	Brilliant Violet 785	M-A251	Biolegend	anti-human	356140
CD3	PE-Cy5	UCHT1	Biolegend	anti-human	300410
CD3	PE-Cy7	UCHT1	Biolegend	anti-human	300420
CD3	PerCP-Cy5.5	UCHT1	Biolegend	anti-human	300430
CD4	Alexa Fluor 700	RPA-T4	Biolegend	anti-human	300526
CD40	APC	5C3	BD Biosciences	anti-human	555591
CD40	Brilliant Violet 711	5C3	Biolegend	anti-human	334334
CD56	PerCP-Cy5.5	5.1H11	Biolegend	anti-human	362506
CD8	Brilliant Violet 650	RPA-T8	Biolegend	anti-human	301042
CD80	Brilliant Violet 421	2D10	Biolegend	anti-human	305222
CD83	Alexa Fluor 647	HB15e	Biolegend	anti-human	305316
CD86	Brilliant Violet 711	IT2.2	Biolegend	anti-human	305440
CD86	PE-Dazzle594	IT2.2	Biolegend	anti-human	305434
HLA-ABC	Brilliant Violet 510	W6/32	Biolegend	anti-human	311436
HLA-DR	Alexa Fluor 700	L243	Biolegend	anti-human	307626
HLA-DR	PE-Cy7	L243	Biolegend	anti-human	307616
IFN-ɣ	PE	4S.B3	Biolegend	anti-human	502509
PD1	Brilliant Violet 711	EH12.2H7	Biolegend	anti-human	329928
PDL1	PE	29E.2A3	Biolegend	anti-human	329706
TNF	Alexa Fluor 647	MAb11	Biolegend	anti-human	502916
Axl	APC	MAXL8DS	eBioScience	anti-mouse	17108482
CD103	PE	2e7	Biolegend	anti-mouse	121406
CD11c	Alexa Fluor 700	N418	Biolegend	anti-mouse	117319
CD11c	PE-Cy7	N418	Biolegend	anti-mouse	117317
CD11c	PE	HL3	BD Biosciences	anti-mouse	557401
CD11c	Alexa Fluor 532	N418	eBioScience	anti-mouse	58011480
CD11c	FITC	HL3	BD Biosciences	anti-mouse	553801
CD127	APC-eFluor780	A7R34	eBioScience	anti-mouse	47127182
CD132	PE	TUGm2	Biolegend	anti-mouse	132306
CD16.2	Pacific Blue	9E9	Biolegend	anti-mouse	149528
CD169	PE-Dazzle594	3D6.112	Biolegend	anti-mouse	142424
CD197/CCR7	PE-Cy5	4B12	Biolegend	anti-mouse	120114
CD25	Brilliant Violet 785	PC61	Biolegend	anti-mouse	102051
CD25	Brilliant Violet 650	PC61	Biolegend	anti-mouse	102037
CD25	PE	PC61	Biolegend	anti-mouse	102008
CD25	PerCP-eFluor 710	PC61.5	eBioScience	anti-mouse	46025182
CD25	Alexa Fluor 700	PC61	Biolegend	anti-mouse	102024
CD3	PerCP-Cy5.5	145-2C11	Biolegend	anti-mouse	100328

CD3	PE-Cy7	145-2C11	Biolegend	anti-mouse	100320
CD3	Brilliant Violet 421	145-2C11	Biolegend	anti-mouse	100336
CD317	Brilliant Violet 605	927	Biolegend	anti-mouse	127025
CD4	Alexa Fluor 700	RM4-5	Biolegend	anti-mouse	100536
CD4	Brilliant Violet 650	RM4-5	Biolegend	anti-mouse	100546
CD4	Brilliant Violet 785	RM4-5	Biolegend	anti-mouse	100552
CD4	FITC	RM4-5	Biolegend	anti-mouse	100510
CD4	PE	RM4-5	Biolegend	anti-mouse	100512
CD4	PE-Dazzle594	GK1.5	Biolegend	anti-mouse	100455
CD40	PE-Cy5	3/23	Biolegend	anti-mouse	124617
CD40	PE-Cy7	3/23	Biolegend	anti-mouse	124622
CD40	APC	3/23	Biolegend	anti-mouse	124612
CD44	Alexa Fluor 647	IM7	Biolegend	anti-mouse	103018
CD44	PE-Cy5	IM7	Biolegend	anti-mouse	103010
CD45	Brilliant Violet 750	30-F11	Biolegend	anti-mouse	103157
CD45.1	Alexa Fluor 700	A20	Biolegend	anti-mouse	110724
CD45.1	Brilliant Violet 421	A20	Biolegend	anti-mouse	110732
CD49b	Alexa Fluor 647	DX5	Biolegend	anti-mouse	108912
CD49b	PerCP-Cy5.5	DX5	Biolegend	anti-mouse	108915
CD49b	Brilliant Violet 421	DX5	BD Biosciences	anti-mouse	563063
CD62L	Brilliant Violet 570	MEL-14	Biolegend	anti-mouse	104433
CD69	APC	H1.2F3	Biolegend	anti-mouse	104514
CD69	Alexa Fluor 700	H1.2F3	Biolegend	anti-mouse	104539
CD69	Brilliant Violet 510	H1.2F3	Biolegend	anti-mouse	104531
CD8	Alexa Fluor 700	53-6.7	Biolegend	anti-mouse	100730
CD8	Brilliant Violet 480	53-6.7	BD Biosciences	anti-mouse	566096
CD8	Brilliant Violet 711	53-6.7	Biolegend	anti-mouse	100748
CD8	PerCP-Cy5.5	53-6.7	Biolegend	anti-mouse	100734
CD8	Pacific Orange	5H10	Thermofisher	anti-mouse	MCD0830
CD8	BV510	53-6.7	Biolegend	anti-mouse	100751
CD80	Brilliant Violet 421	16-10A1	Biolegend	anti-mouse	104726
CD80	V450	16-10A1	BD Biosciences	anti-mouse	560523
CD80	PE-Cy7	16-10A1	Biolegend	anti-mouse	104734
CD86	Alexa Fluor 700	GL-1	Biolegend	anti-mouse	105024
CD86	Brilliant Violet 650	GL-1	Biolegend	anti-mouse	105036
CD86	Brilliant Violet 421	PO3	BD Biosciences	anti-mouse	740034
Clec9A	Brilliant Violet 711	10B4	BD Biosciences	anti-mouse	744513
CTLA-4	PE-Dazzle594	UC10-4B9	Biolegend	anti-mouse	106318
CXCR3	Brilliant Violet 650	CXCR3-173	Biolegend	anti-mouse	126531
F4/80	Brilliant Violet 421	BM8	Biolegend	anti-mouse	123137
F4/80	Brilliant Violet 711	T45-2342	BD Biosciences	anti-mouse	565612
F4/80	Brilliant Violet 510	BM8	Biolegend	anti-mouse	123135
Foxp3	Alexa Fluor 647	MF-14	Biolegend	anti-mouse	126408
Galectin-9	PerCP-eFluor 710	RG9-35	eBioScience	anti-mouse	46-9211-82
Granzyme B	Alexa Fluor 647	GB11	Biolegend	anti-mouse	515406
H2Kd	PerCP-Cy5.5	SF1-1.1	Biolegend	anti-mouse	116618
I-Ad	Alexa Fluor 647	39-10-8	Biolegend	anti-mouse	115010
I-Ad	FITC	39-10-8	Biolegend	anti-mouse	115006
IFN-ɣ	PE-Cy7	XMG1.2	Biolegend	anti-mouse	505826
IFN-ɣ	PE	XMG1.2	Biolegend	anti-mouse	505808
KLRG1	Brilliant Violet 711	2F1	Biolegend	anti-mouse	138427
Ki67	PerCP-efluor710	SolA15	eBioScience	anti-mouse	46569882
Lag3 (CD223)	PE	C9B7W	Biolegend	anti-mouse	125208

Ly6A/E Sca-1	Alexa Fluor 700	D7	Biolegend	anti-mouse	108142
Ly6C	Alexa Fluor 700	HK1.4	Biolegend	anti-mouse	128024
Ly6C	Brilliant Violet 785	HK1.4	Biolegend	anti-mouse	128041
Ly6c	BV421	HK1.4	Biolegend	anti-mouse	128032
Ly6C	PerCP-Cy5.5	HK1.4	Biolegend	anti-mouse	128012
Ly6G	Brilliant Violet 510	1A8	Biolegend	anti-mouse	127633
Ly6G	Brilliant Violet 570	1A8	Biolegend	anti-mouse	127629
MHC-Ib Qa-2	Alexa Fluor 647	695H1-9-9	Biolegend	anti-mouse	121708
OX40	Brilliant Violet 421	OX86	Biolegend	anti-mouse	119411
PD1	Brilliant Violet 421	29F.1A12	Biolegend	anti-mouse	135217
PD1	Brilliant Violet 605	29F.1A12	Biolegend	anti-mouse	135219
PDL1	Brilliant Violet 711	MIH5	BD Biosciences	anti-mouse	563369
PDL1	PE	MIH7	Biolegend	anti-mouse	155404
TCR-β	Alexa Fluor 700	H57-597	Biolegend	anti-mouse	109224
TCR-β	Brilliant Violet 421	H57-597	Biolegend	anti-mouse	109230
TCR-β	Brilliant Violet 570	H57-597	Biolegend	anti-mouse	109231
TCR-β	PE-Cy7	H57-597	Biolegend	anti-mouse	109222
Tim3	PE-Dazzle594	B8.2C12	Biolegend	anti-mouse	134014
TNF	APC	MP6-XT22	Biolegend	anti-mouse	506308
TNF	Brilliant Violet 711	MP6-XT22	Biolegend	anti-mouse	506349
XCR1	Brilliant Violet 650	ZET	Biolegend	anti-mouse	148220
B220	Brilliant Violet 750	RA3-6B2	Biolegend	anti-mouse/anti-human	103261
B220	Brilliant Violet 421	RA3-6B2	Biolegend	anti-mouse/anti-human	103239
B220	PerCP-Cy5.5	RA3-6B2	Biolegend	anti-mouse/anti-human	103236
B220	Brilliant Violet 711	RA3-6B2	Biolegend	anti-mouse/anti-human	103255
B220	BUV496	RA3-6B2	BD Biosciences	anti-mouse/anti-human	612950
CD11b	APC-Cy7	M1/70	Biolegend	anti-mouse/anti-human	101225
CD11b	APC	M1/70	Biolegend	anti-mouse/anti-human	101211
CD11b	PE	M1/70	Biolegend	anti-mouse/anti-human	101208
Tbet	Brilliant Violet 785	4B10	Biolegend	anti-mouse/anti-human	644835
Tbet	PE	4B10	Biolegend	anti-mouse/anti-human	644810
donkey anti-rabbit IgG	Alexa Fluor 647	Poly4064	Biolegend	anti-rabbit	406414

Supplementary Table 3. RT- qPCR primer sequences (5’ to 3’ end).

Gene	Forward primer sequence	Reverse primer sequence	Species
CCL2	TCGCCTCCATCATGAAAGTC	TTGCATCTGGCTGAGCGAG	Human
CCL5	CGGCACGCCTCGCTGTCATC	GCAAGCAGAAACAGGCAAAT	Human
CXCL10	GGAACCTCCAGTCTCAGCACCA	AGACATCTCTTCTCACCCTTC	Human
IFNB1	CAGCTCCAAGAAAGGACGAAC	GGCAGTGTAACTCTTCTGCAT	Mouse
IFNB1	TCTGGCACAACAGGTAGTAGGC	GAGAAGCACAACAGGAGAGCAA	Human
IL6	CTGCAAGAGACTTCCATCCAG	AGTGGTATAGACAGGTCTGTTGG	Mouse
IL6	AGAGGCACTGGCAGAAAACAAC	AGGCAAGTCTCCTCATTGAATCC	Human
IL10	GGGTTGCCAAGCCTTATCG	TCTCACCCAGGGAATTCAAATG	Mouse
IL1B	CTCGCCAGTGAAATGATGGCT	GTCGGAGATTCGTAGCTGGAT	Human
ISG15	GGTGTCCGTGACTAACTCCAT	TGGAAAGGGTAAGACCGTCCT	Mouse
ISG15	TCCTGGTGAGGAATAACAAGGG	GTCAGCCAGAACAGGTCGTC	Human
MX1	GACCATAGGGGTCTTGACCAA	AGACTTGCTCTTTCTGAAAAGCC	Mouse
MX1	GTTTCCGAAGTGGACATCGCA	GAAGGGCAACTCCTGACAGT	Human
STAT1	TCACAGTGGTTCGAGCTTCAG	GCAAACGAGACATCATAGGCA	Mouse
STAT1	ATGTCTCAGTGGTACGAACTTCA	TGTGCCAGGTACTGTCTGATT	Human
TNF	AGAAACACAAGATGCTGGGACAGT	CCTTTGCAGAACTCAGGAATGG	Mouse
TNF	ATGAGCACTGAAAGCATGATCC	GAGGGCTGATTAGAGAGAGGTC	Human
F	CCTTTCTTCTCTAGCAGTGGGAC	TTGAGGACTGTTGTCGGTGAAGC	LaSota virus
