# Supplementary material for: Expanding cross-presenting dendritic cells enhances oncolytic virotherapy and is critical for long-term anti-tumor immunity
Source: Nat Commun. 2022 Nov 22;13:7149. doi: 10.1038/s41467-022-34791-8 (PMC9684150; doi:10.1038/s41467-022-34791-8)
Supplement: Supplementary file 2 — Reporting Summary [file 41467_2022_34791_MOESM2_ESM.pdf]

nature portfolio

Corresponding author(s): Dr Joshua D Brody, MD
Dr Judit Svensson-Arvelund

Last updated by author(s): Sep 11, 2022

Reporting Summary

Nature Portfolio wishes to improve the reproducibility of the work that we publish. This form provides structure for consistency and transparency in reporting. For further information on Nature Portfolio policies, see our Editorial Policies and the Editorial Policy Checklist.

Statistics

For all statistical analyses, confirm that the following items are present in the figure legend, table legend, main text, or Methods section.

n/a	Confirmed	
☐	☒	The exact sample size (n) for each experimental group/condition, given as a discrete number and unit of measurement
☐	☒	A statement on whether measurements were taken from distinct samples or whether the same sample was measured repeatedly
☐	☒	The statistical test(s) used AND whether they are one- or two-sided<br>*Only common tests should be described solely by name; describe more complex techniques in the Methods section.*
☒	☐	A description of all covariates tested
☐	☒	A description of any assumptions or corrections, such as tests of normality and adjustment for multiple comparisons
☐	☒	A full description of the statistical parameters including central tendency (e.g. means) or other basic estimates (e.g. regression coefficient) AND variation (e.g. standard deviation) or associated estimates of uncertainty (e.g. confidence intervals)
☐	☒	For null hypothesis testing, the test statistic (e.g. F, t, r) with confidence intervals, effect sizes, degrees of freedom and P value noted<br>*Give P values as exact values whenever suitable.*
☒	☐	For Bayesian analysis, information on the choice of priors and Markov chain Monte Carlo settings
☒	☐	For hierarchical and complex designs, identification of the appropriate level for tests and full reporting of outcomes
☒	☐	Estimates of effect sizes (e.g. Cohen's d, Pearson's r), indicating how they were calculated

Our web collection on statistics for biologists contains articles on many of the points above.

Software and code

Policy information about availability of computer code

Data collection	FACS Diva 6 (BD) and Attune NxT Software (ThermoFisher) for conventional flow cytometry<br>SpectroFlo Software (Cytek) for spectral flow cytometry<br>ZEN Black (version 2.3) for microscopy<br>HiSeq 4000 (Illumina), for sequencing<br>Living Image software (PerkinElmer)
Data analysis	Cytobank for flow cytometry<br>FIJI for microscopy<br>HTSeqGenie in BioConductor, for DNAseq and RNAseq<br>Morpheus, https://software.broadinstitute.org/morpheus, for qPCR data<br>GraphPad Prism 9.0

For manuscripts utilizing custom algorithms or software that are central to the research but not yet described in published literature, software must be made available to editors and reviewers. We strongly encourage code deposition in a community repository (e.g. GitHub). See the Nature Portfolio guidelines for submitting code & software for further information.

Data

Policy information about availability of data

All manuscripts must include a data availability statement. This statement should provide the following information, where applicable:

- Accession codes, unique identifiers, or web links for publicly available datasets
- A description of any restrictions on data availability
- For clinical datasets or third party data, please ensure that the statement adheres to our policy

Data Availability:
The datasets supporting the findings presented in this study are available from the corresponding author upon reasonable request. All requests for data and materials will be promptly reviewed by the Icahn School of Medicine at Mount Sinai to verify whether the request is subject to any intellectual property or confidentiality obligations. Patient-related data were generated as part of a clinical trial and may be subject to patient confidentiality. Any data that can be shared will be released via a Material Transfer Agreement.

Human research participants

Policy information about studies involving human research participants and Sex and Gender in Research.

Reporting on sex and gender	Samples from both men and women were used, however, sex and gender analyses were not performed since it was not of relevance for the current study; patient samples were primarily used to validate mechanistic results obtained from mouse data.
Population characteristics	Our research did not involve human research participants, but used samples from lymphoma patients, obtained as part of several clinical trials (NCT01976585, NCT03789097, NCT03305445, NCT03153202). Participants in this study were 53% female and 47% male. The age range was 35-79 years old.
Recruitment	Patients were not specifically recruited for this study
Ethics oversight	Protocols for the treatment of patients, and human sample collection and analysis, were approved by the Mount Sinai Institutional Review Board, and written informed consent was obtained from all patients in accordance with the Declaration of Helsinki. All experiments including human specimens were performed in compliance with the relevant ethical regulations.

Note that full information on the approval of the study protocol must also be provided in the manuscript.

Field-specific reporting

Please select the one below that is the best fit for your research. If you are not sure, read the appropriate sections before making your selection.

☒ Life sciences ☐ Behavioural & social sciences ☐ Ecological, evolutionary & environmental sciences

For a reference copy of the document with all sections, see nature.com/documents/nr-reporting-summary-flat.pdf

Life sciences study design

All studies must disclose on these points even when the disclosure is negative.

Sample size	Sample size was based on our previous experience with similar experiments and publications, in particular "Hammerich et al 2019, Nat Med, 2019 May;25(5):814-824":<br><br>"All experiments were designed for two-cohort comparison, those experiments with more than two cohorts all consider the comparison of the (multiple) experimental cohorts to the control cohort as defined for each experiment. Our primary endpoint for each study is the proportion of mice that appear to be cured of disease (therapeutic experiments) or in which disease (tumor growth) is prevented (prophylactic experiments). In our experience with these and related tumor immunotherapy models, we consider the baseline 'cure rate' to be 0% as these tumors are generally progressive and we consider an explicitly beneficial cure rate to be 50%. For these assumptions 10 mice per group are the smallest number of animals that will allow for a statistically significant result, i.e. for 0/10 versus 5/10, two-tailed P value equals 0.0325, whereas for 0/8 versus 4/8 two-tailed P value equals 0.0769 (not significant). "<br><br>Therefore, we aimed for groups of at least 10 mice for all initial in vivo experiments in order to reach statistical significance. For subsequent experiments where the cure rate for the combined treatment was already known to exceed 50%, a minimum of 8 mice per group were used.
Data exclusions	No data were excluded from analysis
Replication	Results were consistently replicated across experiments as indicated in figure legends.

Randomization	Mice and human samples were randomly allocated between groups.
Blinding	Blinding was not possible, since all groups were treated differently and injected repeatedly, and the same individual was responsible for preparing materials for injection and completing all experimental procedures throughout the experiment.

Reporting for specific materials, systems and methods

We require information from authors about some types of materials, experimental systems and methods used in many studies. Here, indicate whether each material, system or method listed is relevant to your study. If you are not sure if a list item applies to your research, read the appropriate section before selecting a response.

Materials & experimental systems

n/a	Involved in the study
☐	☒ Antibodies
☐	☒ Eukaryotic cell lines
☒	☐ Palaeontology and archaeology
☐	☒ Animals and other organisms
☒	☐ Clinical data
☒	☐ Dual use research of concern

Methods

n/a	Involved in the study
☒	☐ ChIP-seq
☐	☒ Flow cytometry
☒	☐ MRI-based neuroimaging

Antibodies

Antibodies used

Flow cytometry Abs:

Marker // Fluorophore // Clone // Company // Species // Cat#
CD11c // FITC // B-ly6 // BD Biosciences // anti-human // 561355
CD123 // Brilliant Violet 650 // 6H6 // Biolegend // anti-human // 306019
CD14 // Brilliant Violet 570 // M5E2 // Biolegend // anti-human // 301832
CD14 // FITC // M5E2 // Biolegend // anti-human // 982502
CD141 // Brilliant Violet 605 // M80 // Biolegend // anti-human // 344117
CD16 // Alexa Fluor 700 // 3G8 // Biolegend // anti-human // 302026
CD19 // APC // HIB-19 // Biolegend // anti-human // 302212
CD19 // Pacific Blue // HIB-19 // Biolegend // anti-human // 302232
CD19 // PE-Cy7 // HIB-19 // Biolegend // anti-human // 302216
CD19 // PerCP-Cy5.5 // HIB-19 // Biolegend // anti-human // 302230
CD1c // PerCP-eFluor 710 // L161 // eBioScience // anti-human // 46001542
CD20 // PE // 2H7 // BD Biosciences // anti-human // 556633
CD25 // Brilliant Violet 785 // M-A251 // Biolegend // anti-human // 356140
CD3 // PE-Cy5 // UCHT1 // Biolegend // anti-human // 300410
CD3 // PE-Cy7 // UCHT1 // Biolegend // anti-human // 300420
CD3 // PerCP-Cy5.5 // UCHT1 // Biolegend // anti-human // 300430
CD4 // Alexa Fluor 700 // RPA-T4 // Biolegend // anti-human // 300526
CD40 // APC // 5C3 // BD Biosciences // anti-human // 555591
CD40 // Brilliant Violet 711 // 5C3 // Biolegend // anti-human // 334334
CD56 // PerCP-Cy5.5 // 5.1H11 // Biolegend // anti-human // 362506
CD8 // Brilliant Violet 650 // RPA-T8 // Biolegend // anti-human // 301042
CD80 // Brilliant Violet 421 // 2D10 // Biolegend // anti-human // 305222
CD83 // Alexa Fluor 647 // HB15e // Biolegend // anti-human // 305316
CD86 // Brilliant Violet 711 // IT2.2 // Biolegend // anti-human // 305440
CD86 // PE-Dazzle594 // IT2.2 // Biolegend // anti-human // 305434
HLA-ABC // Brilliant Violet 510 // W6/32 // Biolegend // anti-human // 311436
HLA-DR // Alexa Fluor 700 // L243 // Biolegend // anti-human // 307626
HLA-DR // PE-Cy7 // L243 // Biolegend // anti-human // 307616
IFN-ɣ // PE // 4S.B3 // Biolegend // anti-human // 502509
PD1 // Brilliant Violet 711 // EH12.2H7 // Biolegend // anti-human // 329928
PDL1 // PE // 29E.2A3 // Biolegend // anti-human // 329706
TNF // Alexa Fluor 647 // MAb11 // Biolegend // anti-human // 502916
Axl // APC // MAXL8DS // eBioScience // anti-mouse // 17108482
CD103 // PE // 2e7 // Biolegend // anti-mouse // 121406
CD11c // Alexa Fluor 700 // N418 // Biolegend // anti-mouse // 117319
CD11c // PE-Cy7 // N418 // Biolegend // anti-mouse // 117317
CD11c // PE // HL3 // BD Biosciences // anti-mouse // 557401
CD11c // Alexa Fluor 532 // N418 // eBioScience // anti-mouse // 58011480
CD11c // FITC // HL3 // BD Biosciences // anti-mouse // 553801
CD127 // APC-eFluor780 // A7R34 // eBioScience // anti-mouse // 47127182
CD132 // PE // TUGm2 // Biolegend // anti-mouse // 132306
CD16.2 // Pacific Blue // 9E9 // Biolegend // anti-mouse // 149528
CD169 // PE-Dazzle594 // 3D6.112 // Biolegend // anti-mouse // 142424

CD197/CCR7 // PE-Cy5 // 4B12 // Biolegend // anti-mouse // 120114
CD25 // Brilliant Violet 785 // PC61 // Biolegend // anti-mouse // 102051
CD25 // Brilliant Violet 650 // PC61 // Biolegend // anti-mouse // 102037
CD25 // PE // PC61 // Biolegend // anti-mouse // 102008
CD25 // PerCP-eFluor 710 // PC61.5 // eBioScience // anti-mouse // 46025182
CD25 // Alexa Fluor 700 // PC61 // Biolegend // anti-mouse // 102024
CD3 // PerCP-Cy5.5 // 145-2C11 // Biolegend // anti-mouse // 100328
CD3 // PE-Cy7 // 145-2C11 // Biolegend // anti-mouse // 100320
CD3 // Brilliant Violet 421 // 145-2C11 // Biolegend // anti-mouse // 100336
CD317 // Brilliant Violet 605 // 927 // Biolegend // anti-mouse // 127025
CD4 // Alexa Fluor 700 // RM4-5 // Biolegend // anti-mouse // 100536
CD4 // Brilliant Violet 650 // RM4-5 // Biolegend // anti-mouse // 100546
CD4 // Brilliant Violet 785 // RM4-5 // Biolegend // anti-mouse // 100552
CD4 // FITC // RM4-5 // Biolegend // anti-mouse // 100510
CD4 // PE // RM4-5 // Biolegend // anti-mouse // 100512
CD4 // PE-Dazzle594 // GK1.5 // Biolegend // anti-mouse // 100455
CD40 // PE-Cy5 // 3/23 // Biolegend // anti-mouse // 124617
CD40 // PE-Cy7 // 3/23 // Biolegend // anti-mouse // 124622
CD40 // APC // 3/23 // Biolegend // anti-mouse // 124612
CD44 // Alexa Fluor 647 // IM7 // Biolegend // anti-mouse // 103018
CD44 // PE-Cy5 // IM7 // Biolegend // anti-mouse // 103010
CD45 // Brilliant Violet 750 // 30-F11 // Biolegend // anti-mouse // 103157
CD45.1 // Alexa Fluor 700 // A20 // Biolegend // anti-mouse // 110724
CD45.1 // Brilliant Violet 421 // A20 // Biolegend // anti-mouse // 110732
CD49b // Alexa Fluor 647 // DX5 // Biolegend // anti-mouse // 108912
CD49b // PerCP-Cy5.5 // DX5 // Biolegend // anti-mouse // 108915
CD49b // Brilliant Violet 421 // DX5 // BD Biosciences // anti-mouse // 563063
CD62L // Brilliant Violet 570 // MEL-14 // Biolegend // anti-mouse // 104433
CD69 // APC // H1.2F3 // Biolegend // anti-mouse // 104514
CD69 // Alexa Fluor 700 // H1.2F3 // Biolegend // anti-mouse // 104539
CD69 // Brilliant Violet 510 // H1.2F3 // Biolegend // anti-mouse // 104531
CD8 // Alexa Fluor 700 // 53-6.7 // Biolegend // anti-mouse // 100730
CD8 // Brilliant Violet 480 // 53-6.7 // BD Biosciences // anti-mouse // 566096
CD8 // Brilliant Violet 711 // 53-6.7 // Biolegend // anti-mouse // 100748
CD8 // PerCP-Cy5.5 // 53-6.7 // Biolegend // anti-mouse // 100734
CD8 // Pacific Orange // 5H10 // Thermofisher // anti-mouse // MCD0830
CD8 // BV510 // 53-6.7 // Biolegend // anti-mouse // 100751
CD80 // Brilliant Violet 421 // 16-10A1 // Biolegend // anti-mouse // 104726
CD80 // V450 // 16-10A1 // BD Biosciences // anti-mouse // 560523
CD80 // PE-Cy7 // 16-10A1 // Biolegend // anti-mouse // 104734
CD86 // Alexa Fluor 700 // GL-1 // Biolegend // anti-mouse // 105024
CD86 // Brilliant Violet 650 // GL-1 // Biolegend // anti-mouse // 105036
CD86 // Brilliant Violet 421 // PO3 // BD Biosciences // anti-mouse // 740034
Clec9A // Brilliant Violet 711 // 10B4 // BD Biosciences // anti-mouse // 744513
CTLA-4 // PE-Dazzle594 // UC10-4B9 // Biolegend // anti-mouse // 106318
CXCR3 // Brilliant Violet 650 // CXCR3-173 // Biolegend // anti-mouse // 126531
F4/80 // Brilliant Violet 421 // BM8 // Biolegend // anti-mouse // 123137
F4/80 // Brilliant Violet 711 // T45-2342 // BD Biosciences // anti-mouse // 565612
F4/80 // Brilliant Violet 510 // BM8 // Biolegend // anti-mouse // 123135
Foxp3 // Alexa Fluor 647 // MF-14 // Biolegend // anti-mouse // 126408
Galectin-9 // PerCP-eFluor 710 // RG9-35 // eBioScience // anti-mouse // 46-9211-82
Granzyme B // Alexa Fluor 647 // GB11 // Biolegend // anti-mouse // 515406
H2Kd // PerCP-Cy5.5 // SF1-1.1 // Biolegend // anti-mouse // 116618
I-Ad // Alexa Fluor 647 // 39-10-8 // Biolegend // anti-mouse // 115010
I-Ad // FITC // 39-10-8 // Biolegend // anti-mouse // 115006
IFN-ɣ // PE-Cy7 // XMG1.2 // Biolegend // anti-mouse // 505826
IFN-ɣ // PE // XMG1.2 // Biolegend // anti-mouse // 505808
KLRG1 // Brilliant Violet 711 // 2F1 // Biolegend // anti-mouse // 138427
Ki67 // PerCP-efluor710 // SolA15 // eBioScience // anti-mouse // 46569882
Lag3 (CD223) // PE // C9B7W // Biolegend // anti-mouse // 125208
Ly6A/E Sca-1 // Alexa Fluor 700 // D7 // Biolegend // anti-mouse // 108142
Ly6C // Alexa Fluor 700 // HK1.4 // Biolegend // anti-mouse // 128024
Ly6C // Brilliant Violet 785 // HK1.4 // Biolegend // anti-mouse // 128041
Ly6c // BV421 // HK1.4 // Biolegend // anti-mouse // 128032
Ly6C // PerCP-Cy5.5 // HK1.4 // Biolegend // anti-mouse // 128012
Ly6G // Brilliant Violet 510 // 1A8 // Biolegend // anti-mouse // 127633
Ly6G // Brilliant Violet 570 // 1A8 // Biolegend // anti-mouse // 127629
MHC-Ib Qa-2 // Alexa Fluor 647 // 695H1-9-9 // Biolegend // anti-mouse // 121708
OX40 // Brilliant Violet 421 // OX86 // Biolegend // anti-mouse // 119411
PD1 // Brilliant Violet 421 // 29F.1A12 // Biolegend // anti-mouse // 135217
PD1 // Brilliant Violet 605 // 29F.1A12 // Biolegend // anti-mouse // 135219
PDL1 // Brilliant Violet 711 // MIH5 // BD Biosciences // anti-mouse // 563369
PDL1 // PE // MIH7 // Biolegend // anti-mouse // 155404
TCR-β // Alexa Fluor 700 // H57-597 // Biolegend // anti-mouse // 109224
TCR-β // Brilliant Violet 421 // H57-597 // Biolegend // anti-mouse // 109230
TCR-β // Brilliant Violet 570 // H57-597 // Biolegend // anti-mouse // 109231

	TCR-β // PE-Cy7 // H57-597 // Biolegend // anti-mouse // 109222<br>Tim3 // PE-Dazzle594 // B8.2C12 // Biolegend // anti-mouse // 134014<br>TNF // APC // MP6-XT22 // Biolegend // anti-mouse // 506308<br>TNF // Brilliant Violet 711 // MP6-XT22 // Biolegend // anti-mouse // 506349<br>XCR1 // Brilliant Violet 650 // ZET // Biolegend // anti-mouse // 148220<br>B220 // Brilliant Violet 750 // RA3-6B2 // Biolegend // anti-mouse/anti-human // 103261<br>B220 // Brilliant Violet 421 // RA3-6B2 // Biolegend // anti-mouse/anti-human // 103239<br>B220 // PerCP-Cy5.5 // RA3-6B2 // Biolegend // anti-mouse/anti-human // 103236<br>B220 // Brilliant Violet 711 // RA3-6B2 // Biolegend // anti-mouse/anti-human // 103255<br>B220 // BUV496 // RA3-6B2 // BD Biosciences // anti-mouse/anti-human // 612950<br>CD11b // APC-Cy7 // M1/70 // Biolegend // anti-mouse/anti-human // 101225<br>CD11b // APC // M1/70 // Biolegend // anti-mouse/anti-human // 101211<br>CD11b // PE // M1/70 // Biolegend // anti-mouse/anti-human // 101208<br>Tbet // Brilliant Violet 785 // 4B10 // Biolegend // anti-mouse/anti-human // 644835<br>Tbet // PE // 4B10 // Biolegend // anti-mouse/anti-human // 644810<br>donkey anti-rabbit IgG // Alexa Fluor 647 // Poly4064 // Biolegend // anti-rabbit // 406414<br><br>All mouse and human surface antibodies were used at a dilution of 1:400 and 1:200, respectively, and for intracellular staining, mouse and human antibodies were used at a 1:200 or 1:100 dilution, respectively.<br><br>Microscopy Abs:<br>Anti-GFP-AlexaFluor 488 (clone FM264G), anti-CD8-AlexaFluor 647 (clone 53-6.7), anti-CD11c-AlexaFluor 594 (clone N418), anti-CD45.1-BV421 (clone A20), from Biolegend, or rabbit anti-cleaved caspase-3 (Asp175) (5A1E, Cell Signaling). All antibodies were used at a 1:200 dilution, except anti-cleaved caspase-3 that was used at a 1:500 dilution.<br><br>In vivo Abs:<br>Anti-mouse IFNAR (clone MAR1-5A3; BioXCell), anti-CD8 (2.43; BioXCell), anti-CD4 (GK1.5; BioXCell).
Validation	All anti-mouse, anti-human, and secondary anti-rabbit antibodies were validated by the manufacturers. Validation statements can be found in the manufacturers' websites (catalogue numbers are provided in Supplementary Table 2)

Eukaryotic cell lines

Policy information about cell lines and Sex and Gender in Research

Cell line source(s)	A20 lymphoma cells were purchased from ATCC (ATCC TIB-208); A20-GFP, A20-mCherry and A20-mCherry-Luciferase were generated in-house using lentiviral transduction; A20-GFP-b2m-/- cells were generated in-house using the Crispr/Cas9 system, and SUDHL4 cells (originally purchsed from DSMZ, ACC 495) were a gift from Dr. David Dominguez-Sola (Mount Sinai). Vero cells (African green monkey kidney epithelial cells) were purchased from ATCC (CCL-81).
Authentication	None of the cell lines used were authenticated
Mycoplasma contamination	All cell lines tested negative for mycoplasma
Commonly misidentified lines<br>(See ICLAC register)	Not used

Animals and other research organisms

Policy information about studies involving animals; ARRIVE guidelines recommended for reporting animal research, and Sex and Gender in Research

Laboratory animals	Mice: Balb/c wt, Balb/c-Batf3-/-, Balb/c-JEDI, male and female, all 8-12 weeks
Wild animals	The study did not involve wild animals
Reporting on sex	We have used both males and females for in vivo and in vitro murine A20 lymphoma experiments. We have previously used both males and females achieving similar results.
Field-collected samples	The study did not involve samples collected from the field
Ethics oversight	All experiments were reviewed and approved by the Institutional Animal Care and Use Committee of the Icahn School of Medicine at Mount Sinai.

Note that full information on the approval of the study protocol must also be provided in the manuscript.

Flow Cytometry

Plots

Confirm that:

☒ The axis labels state the marker and fluorochrome used (e.g. CD4-FITC).

☒ The axis scales are clearly visible. Include numbers along axes only for bottom left plot of group (a 'group' is an analysis of identical markers).

☒ All plots are contour plots with outliers or pseudocolor plots.

☒ A numerical value for number of cells or percentage (with statistics) is provided.

Methodology

Sample preparation	Tumors, spleens and lymph nodes were homogenized by forcing the tissue through a 70µm nylon mesh. Cell suspensions were pelleted at 500xg for 5 minutes at 4°C and red blood cells were lysed with Pharm Lyse lysing buffer (BD). Tumor cell suspensions were then depleted of tumor cells by magnetic separation using CD19-nanobeads according to the manufacturer's protocol (Mojosort, Biolegend). Cells were stored at 4 °C until further usage.
Instrument	LSR Fortessa (BD), Attune (ThermoFisher Scientific) or Aurora (Cytek).<br>Cell sorting was performed on a FACSAria (BD Biosciences).
Software	FACS Diva 6 (BD) and Attune NxT Software (ThermoFisher) for conventional flow cytometry<br>SpectroFlo Software (Cytek) for spectral flow cytometry.<br>Cytobank for data analysis
Cell population abundance	Purity of post-sort samples was typically 95% or higher as determined by flow cytometry
Gating strategy	Cells were first gated based on FSC/SCC, doublets were excluded using FSC-A vs FSC-W (plus SSC-A vs SSC-H for FACS-sorting), cells were then gated on live cells using a viability dye, followed by cell type-specific gating using fluorescently labeled antibodies. Example gating strategies are shown in Supplemental Fig 9.

☒ Tick this box to confirm that a figure exemplifying the gating strategy is provided in the Supplementary Information.
